# Aerospace-foraging bats eat seasonably across varying habitats

**DOI:** 10.1038/s41598-023-46939-7

**Published:** 2023-11-10

**Authors:** Joxerra Aihartza, Nerea Vallejo, Miren Aldasoro, Juan L. García-Mudarra, Urtzi Goiti, Jesus Nogueras, Carlos Ibáñez

**Affiliations:** 1 Department of Zoology and Animal Cell Biology, University of the Basque Country UPV/EHU, Sarriena s/n, 48940 Leioa, The Basque Country Spain; 2 Estación Biológica de Doñana (CSIC), P.O. Box 1056, 41080 Sevilla, Spain

**Keywords:** Ecology, Zoology, Ecology

## Abstract

Recent research has confirmed the efficiency of insectivorous bats as pest suppressors, underlining the ecological services they offer in agroecosystems. Therefore, some efforts try to enhance bat foraging in agricultural landscapes by acting upon environmental factors favouring them. In this study, we monitored a *Miniopterus schreibersii* colony, in the southern Iberian Peninsula. We intensively sampled their faeces and analysed them by metabarcoding to describe how the bent-winged bat diet would change with time, and to test whether their most-consumed prey would seasonally depend on different landscapes or habitats. Our results confirm that *M. schreibersii* are selective opportunist predators of moths, dipterans, mayflies, and other fluttering insects, shifting their diet to temporary peaks of prey availability in their foraging range, including both pest and non-pest insects. Supporting our hypothesis, throughout the year, *M. schreibersii* consume insects linked to diverse open habitats, including wetlands, grassland, diverse croplands, and woodland. The importance of each prey habitat varies seasonally, depending on their insect phenology, making bats indirectly dependent on a diverse landscape as their primary prey source. Bats' predation upon pest insects is quantitatively high, consuming around 1610 kg in 5 months, of which 1467 kg correspond to ten species. So, their suppression effect may be relevant, mainly in patchy heterogeneous landscapes, where bats' foraging may concentrate in successive outbursts of pests, affecting different crops or woodlands. Our results stress that to take advantage of the ecosystem services of bats or other generalist insectivores, keeping the environmental conditions they require to thrive, particularly a heterogeneous landscape within the colony's foraging area, is crucial.

## Introduction

Insectivorous bats prey upon a wide variety of arthropods, consuming between 30 and 80% of their body mass each night due to their high metabolic rate^1,2^. Not surprisingly, even before the morphological techniques used in diet studies could identify the consumed prey beyond the family level, scientists foresaw the likely beneficial effect of these mammals' predatory behaviour in the fight against pest arthropods^3–6^. This idea flourished long before the term Ecosystem Services was coined^7^.

The recent development of molecular methods based on DNA metabarcoding and High Throughput Sequencing (HTS) offers superior detection ability and accurate identification of the consumed prey to the species level [e.g.,^8–10^]. During the last decade, these methods have been widely applied to broad studies of trophic ecology^11^ and provided comprehensive diet descriptions for a vast list of animals [e.g.,^12–17^].

In the case of insectivorous bats, many molecular studies have described their food habits in detail [e.g.,^18–25^]. Moreover, several pieces of research have confirmed the predation of bats upon a wide array of agroforestry pests, underlining the ecological services that these mammals offer as consumers—and likely suppressors—of them [e.g.,^26–32^]. Not surprisingly, molecular diet studies on bats in agroecosystems have become an up-and-coming research field. These studies delve deeper into the ecological relationships between those mammals and the environment. As applied research, the authors aim to take advantage of the ecosystem services bats most likely provide [e.g.,^33–35^].

Insect pests have increased during the last century due to the intensification of agriculture and invasive forest management practices^36^. In the previous decades, though, regulating of chemical pesticides in agriculture has become tighter while pests are rapidly developing resistance to them^37^. In this scenario, the biological suppression of agroforestry pests appears as a suitable solution^38,39^. Many authors have supported the efficiency of bats as pest controllers^31,40–44^. Moreover, Boyles et al.^33^ valued the ecosystem services bats provided at $22.9 billion per year on agroecosystems in the United States; Wanger et al.^44^ estimated $1.2 million yearly in rice plantations in Thailand. Thus, some efforts have tried to promote ecosystem services by bats by providing supplementary roost availability or studying which environmental factors favour bat foraging in agricultural landscapes. For example, after the installation of bat boxes in rice paddies and their occupation by soprano pipistrelles (*Pipistrellus pygmaeus*), the striped rice borer moth (*Chilo suppressalis*) declined below the chemical treatment threshold at Delta del Ebre in Catalonia^42^.

Moths comprise major agricultural pests damaging crops worldwide^45^. They are the primary prey of many insectivorous bats, including several moth specialist species: e.g., *Rhinolophus euryale* ^19,46^, *Tadarida teniotis*^47^, *Plecotus* sp.^18,48^, or the open space forager *Miniopterus schreibersii*^26^.

As is well known, insectivorous bats are highly adaptive and respond to a wide variety of prey, adjusting their diet to changes in availability [e.g.,^30,49,50^]. Most agroforestry pest insects occur in cyclic outbreaks^51^, which may be a consequence of their population density dynamics^52–55^, environmental pressures^56,57^, or even an answer to global change^58^. Therefore, bats must have other trophic resources available throughout time, apart from pests, to set colonies that will thrive. Furthermore, bats will shift their foraging grounds toward those with higher prey availability^49^. There, they often consume prey that originated outside the habitats where they are hunted^19^. Consequently, management measures addressing bats' foraging requirements should also consider the prey’s ecological requirements throughout their life stages.

The bent-winged bat *M. schreibersii*, is a cave-dwelling bat responsible for the largest colonies in Europe: up to 60,000 individuals were reported roosting in the same place in Bulgaria^59^. They perform annual migrations to roosts of ideal microclimatic conditions^60^. Due to their eco-morphological characteristics and high-speed flight^61^, nightly, the bent-winged bats fly up to 30 km from their roost to forage, showing one of the most extensive home ranges in European bats^62^. Despite such foraging ranges, they use proportionally small individual hunting areas, suggesting an uneven distribution of resources^62^.

The traits mentioned above make bent-winged bats optimal candidates to efficiently suppress pest insects in agroecosystems. Accordingly, an extensive molecular diet study at a continental scale^26^ showed that *M. schreibersii* consumes up to 44 crop insect species in Europe, likely being a valuable asset for biological pest suppression in various agricultural productions. The study addressed in detail neither the seasonal variation of the species' diet in the same locality nor the primary habitat of the main prey they consumed at the time or locality.

In this study, we analysed the changes in the diet of a large colony of *M. schreibersii* in an agricultural landscape across their active season in the Iberian Peninsula, paying attention to the seasonal variations in diet and the habitats on which the most consumed preys depend. Our primary hypotheses were: (1) The bent-winged bat diet will change with time, showing a seasonally shifting most-consumed prey sequence; (2) Those most-consumed prey will depend on different landscapes or habitats, causing seasonal changes in the required environmental resources for the bats. And (3), we aim to identify the main pest insects these bats prey upon in this agricultural landscape and quantitatively assess the magnitude order in which such consumption might move.

## Material and methods

### Study area

We conducted this study in a roost of *M. schreibersii* located at the Southernmost point of the Iberian Peninsula, near the cities El Puerto de Santa Maria and Jerez de la Frontera (Andalusia), approximately 15 km from the coast. Las Colmenas (90 m asl) is an artificial cave built to be an underground sandstone quarry in Sierra de San Cristobal range. Stone exploitation began in the Phoenician and Roman periods and peaked in the XVI and XVII centuries. The galleries, used as an ammunition dump in the second half of the XX century, fell into disuse at the beginning of the XXI. Since 2015, it has been included in Special Areas of Conservation (SAC No. ES6120030) due to the populations of cave-dwelling bats *Myotis myotis, Myotis blythii, M. schreibersii* roosting during the breeding season^63^.

**Figure 1:**
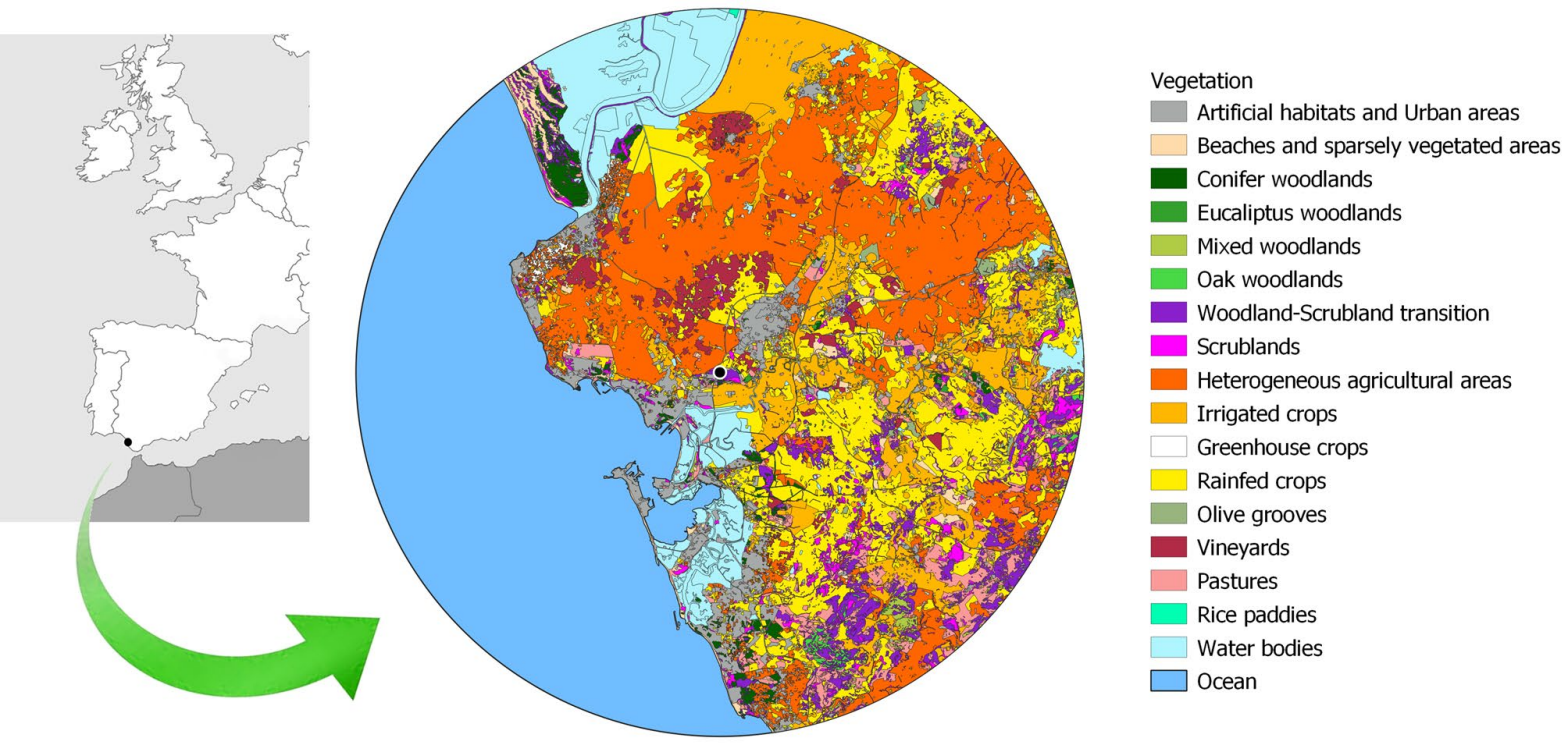
Study area: main habitat and land cover units within 40 km around the studied bat roost. The map was built with Qgis v 3.24^65^, based un public maps by Junta de Andalucía^66^.

The SAC Sierra de San Cristóbal holds 47.95 ha around a hill with 129 m asl. The vegetation is Mediterranean scrubland, where broom prevails (*Retama sphaerocarpa*), interspersed with scattered pine *Pinus pinea* ^64^. Highly modified croplands and urban areas encircle it. In a 40 km radius, sea occupies approximately 30% of the surface (Fig. 1). Diverse croplands cover two-thirds of the land surface; natural vegetation (pastures, scrubland, and woodland), marshes and dams, and urbanized and industrial lands cover the remaining third (Table 1). The landscape comprises smooth hills, with about 90% of the surface below 100 m asl and the highest at 412 m at the Eastern zone.

**Table 1:** Landscape use within a 40 km radius from the roost.

Landscape use	Surface (ha)
Cropland	217,834
Natural vegetation, pastures, scrubland and woodland	65,953
Marshes and dams	39,342
Urbanized areas	27,322
Total land surface	350,451
Sea surface	152,204
Total	502,655

Regarding the croplands, 150,000 ha are devoted to rainfed annual crops, primarily cereals (wheat) and, to a lesser extent, sunflower. Non-irrigated wood crops comprise 15,000 ha, including 12,000 ha of vineyards and 3,000 ha of olive groves. Irrigated crops occupy 50,000 ha, mostly cotton and, to a lesser extent, corn, sunflower, tomato, rice and other horticultural ones. With less than 10,000 ha, pine woodland ranges from high density to scattered trees. The majority is located further than 25 km from the roost, with a main continuous patch in the North (within Doñana National Park) and smaller scattered patches closer to the seashore. Holm oak (*Quercus ilex*) and/or cork oak (*Quercus suber*) woodlands comprise around 6,000 ha in scattered patches, mainly further than 20 km from the roost, in the SE quadrant. Below Sierra de San Cristobal, the Guadalete River, its estuary and marshes (Bahía de Cádiz Natural Parc) offer diverse wetland habitats suitable for different insects (mostly Diptera and mayflies), which are putative prey for bats.

### Sample collection

The *M. schreibersii* colony occupies the same roost from April to November, so sampling was carried out every two weeks from early May to early November 2018.

Each sampling night, we monitored the colony size, video-recording the emergence of bats by a near IR camera (SONY Handycam HDR-PJ780VE), lighted with an infrared torch Raytec VAR-I2-1 and connected to a frequency-division ultrasound detector Pettersson Elektronik D-240 as a microphone. The cave has a single entrance at ground level; the camera was set 8 m away from it, laterally to the bats' flight-way, fully covering the entrance. Recordings began after the first bat emerged (usually about 15 min after sunset) and continued for one hour, ensuring the full emergence was captured. Video and ultrasound recordings were analysed manually, simultaneously playing the video and ultrasound spectrograms (with BatSound v. 4.0, Pettersson Elektronik).

To collect the faecal samples, bats were captured entering the roost after the night foraging, using mist nets, and kept in individual cloth bags until they defecated. Ten samples of eight to ten faecal pellets each, taken from ten individual bats, were collected each night and stored for analysis; these yielded 140 samples in 14 nights. Afterwards, bats were immediately released to their roost to minimise their stress.

### Ethics statement

We performed the captures under license from the Autonomous Government of Andalusia (resolution of 15/4/2019 by Dirección General de Medio Natural, Biodiversidad y Espacios Protegidos). The methods were performed with protocols that followed the guidelines for the treatment of animals in research and teaching^67^, in compliance with the ARRIVE guidelines, and approved by the Ethical committee at the EBD–CSIC (Estación Biologica de Doñana, Consejo Superior de Investigaciones Científicas).

### DNA extraction, PCR amplification and sequencing

Extractions were performed in the Biological Station of Doñana (EBD-CSIC, Spain) using the DNA extraction kit DNeasy PowerSoil Kit (Qiagen), following the manufacturer’s instructions. We included extraction blanks in every extraction round. All samples were amplified with a combination of two primers that target the COI gene: (1) Zeale ZBJ-ArtR2c and ZBJ-ArtF1c, in the future called ‘Zeale’^68^; and (2) modified LepF1 and EPT-long-univR, from now on ‘Gillet’^69^. These two primer pairs complement each other and allow the detection of a broader spectrum of prey species^70,71^ compared to using one primer pair alone. PCR products migrated in agarose gel electrophoresis to test the success of the amplification process. Subsequently, a second PCR reaction was performed to attach a unique combination of tags and Illumina sequencing adapters to each amplicon using the Nextera XT Index Kit^72^. Finally, samples were pooled and sequenced using Illumina MiSeq technology. DNA library construction and sequencing processes were done at the Genomics and Proteomics General Service (SGIker) of the University of the Basque Country.

### Sequence analysis and library building

Using *Usearch* v.10^73^, paired-end reads were merged in those sequences retrieved from Illumina. Then, a quality filter was applied to discard possible sequencing errors, using the Q30 quality value as a threshold. Reads were then demultiplexed according to primer sequences, primers were trimmed, and the remaining sequences were selected according to the appropriate length for each marker using *Cutadapt*^74^. Singleton sequences were set aside, and the remaining sequences were clustered into ZOTUs (Zero-Radius Operational Taxonomic Units), using the *-unoise3* command in *Usearch*. This step implements denoising, i.e., error correction, to the sequence clustering step and maintains better biological resolution^75^. Then, for each sample, the ZOTUs with frequencies lower than 1% were removed using *Usearch*’s *-otutab_norm* and *-otutab_trim* commands. For the Gillet dataset, the first ZOTU, which belonged to the predator, was removed for this step because the relative read abundance of this ZOTU was considerably higher than the rest and limited the recoverable diversity in each sample. Finally, we compared each ZOTU against the online databases BOLD Systems and GenBank, following identification criteria by Clare et al.^9^ and Vesterinen et al.^76^.

### Diet description and analysis

All statistical analyses were conducted in R version 4.0.4^77^. The percentage of occurrence of each prey item was weighted by the number of prey items identified in that sample (wPOO:^78^).

We used distance-based redundancy analysis (db-RDA:^79^) to study the multivariate relationship of the diet with each of the sampling dates, which enabled us to select up to 26 prey species: the consumption of 22 of them was correlated with a specific sampling day, while four were often consumed but not related to seasonality. This analysis is further described in Supplementary Material 1. The consumption of the 22 species important for the constrained axes was modelled against date with Generalized Additive Mixed Models (GAMM) using function *gamm* in package *mgcv*^80^. We calculated the wPOO values of the 22 prey species per sampling date, log-transformed them, and modelled them as a day-of-the-year non-linear function representing the sampling date. Since the consumption of each species is different throughout the season, we should model a smoother for each species. Nonetheless, there needs to be more data points to do so reliably. Instead, an exploratory analysis showed that species could be paired together as their consumption followed similar trends. Therefore, the final model has eleven groups of species and a random intercept, which allows for different variances for each prey species. Given the temporality of the data, we considered adding a temporal correlation structure to the model. Still, based on Akaike’s information criterion (AIC) it did not improve, leaving it out of the final model.

The final model formulation is the following:

1 $$\begin{aligned} log\left( {wPOO_{sp, date} + 1} \right) & = \alpha_{pair} + f_{group} \left( {DayofYear} \right) + \varepsilon_{group,date} \\ & \;\;\;\varepsilon_{group,date} \sim N\left( {0, \sigma_{group}^{2} } \right) \\ \end{aligned}$$

where:

*sp*, each prey species added to the model.

*date*, day of the year the sampling was conducted.

*group*, groups of prey species modelled together because they show similar trends.

For smoothing curves, p-values near 0.05 should be interpreted cautiously^81^, so we set the smoother significance threshold at a p-value of 0.01.

### Characterisation of prey’s source habitats

We gathered information on the habitat preferences of the 26 prey species selected from the db-RDA analysis from several bibliographic sources and online databases (references available in Supplementary Material 2). We then summarised this information in a binary table composed of vegetation characteristics (15 categories) and vegetation density (3 classes) based on the habitat types available within 40 km around the colony.

We built a sampling date × habitat matrix using the prey × habitat binary matrix and the wPOO values for each prey (Supplementary Material l 3a). The prey × habitat table was first weighted to account for the differences in coverage of the habitats around the colony. Then, we multiplied it with the wPOO matrix, resulting in a sampling date × habitat matrix (Supplementary material 3b). Finally, average values for each habitat were calculated across all sampling dates to get a profile of the preferred habitats of the most consumed prey of *M. schreibersii,* accounting for their consumption—hereafter referred to as “prey’s source habitats”—. These “prey’s source habitats” were compared against the area of different types of habitats available around the colony—hereafter “available habitats”—using a Chi-square test of independence and simulated p-values by Monte Carlo test (2000 replicates) due to the low frequencies of some habitat categories.

### Pest insects’ classification

First, we checked all the species to which the ZOTUs had been ascribed in the European and Mediterranean Plant Protection Organization (EPPO) Global Database^82^ to test which of them had been categorised as a pest in different degrees. Second, we classified them more conservatively following the Ministry of Agriculture of Spain (Supplementary Material 4), determining which major or minor pests were considered for different crops or woodlands.

### A quantitative approach to pest insect consumption

To get a first quantitative approach to the pest insect consumption by the studied bat colony within their foraging grounds, we estimated the consumption of each of the selected pest items in every sampling date using the following formula:

2 $${\text{Consumption}}_{{{\text{Pest}}}} \left( {\text{g}} \right) \, = {\text{ c }}*{\text{ meanweight }}*{\text{ colsize }}*{\text{ wPOO}}_{{{\text{Pest}}}}$$

where:

*c*: Kurta et al.^1^ estimated that in the temperate insectivorous bat *Myotis lucifugus,* individuals should daily eat about 70% of their body weight in insects to fulfil their requirements during pregnancy and lactation.

*meanweight*: 340 individual *M. schreibersii* bats (170 males and 170 females) captured in the study area in June–August 2018 weighted 11.92 g on average (SD = 0.75).

*colsize:* We monitored the colony size each sampling session during the study—above—.

*wPOOPest:* We relied on wPOO^78,83^ as the best proxy for translating molecular diet results to quantitative assessment of diet composition.

Based on the data collected every fifteen days during 2018, we predicted the consumption of relevant pest species every day throughout the sampling season using a LOESS smoother (span = 0.5). Based on that and weighting the total consumption of each pest species with the surface of their habitat in the study area, we calculated the predation intensity upon them, in kg/ha.

### Ethical approval

The authors performed the captures under license from the Autonomous Government of Andalusia (resolution of 15/4/2019 by Dirección General de Medio Natural, Biodiversidad y Espacios Protegidos) with protocols that followed the published guidelines for the treatment of animals in research and teaching (66).

## Results

**Figure 2:**
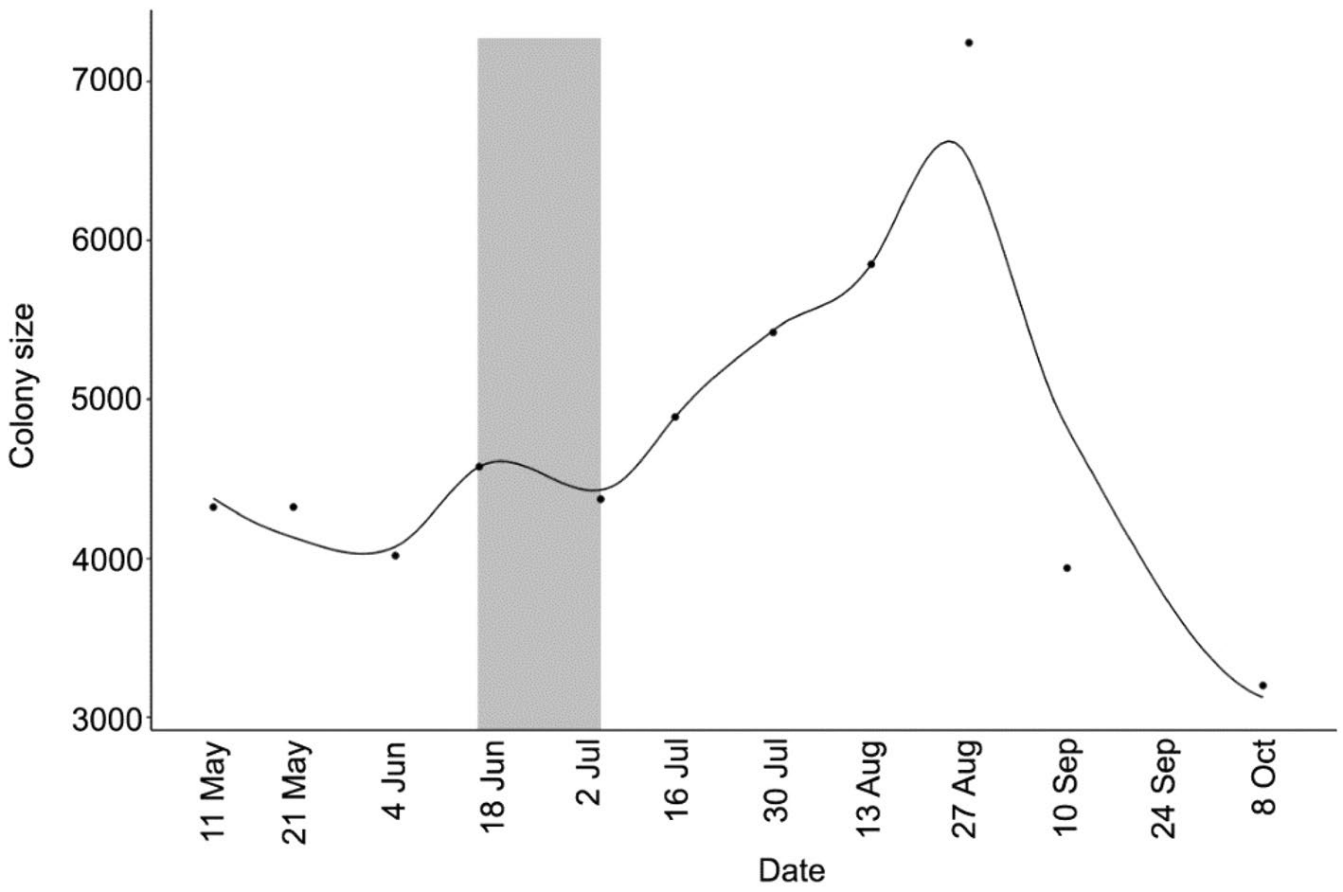
Variation of the *M. schreibersii* colony size during the study period. The animals were counted by video recording during the emergence at dusk. The points represent the observed values; the trend is represented with LOESS smoother. The grey square indicates presence of lactating females.

The colony size ranged from 3200 to 7244 individuals during the study period, with a minimum size in October and a maximum in late August (Fig. 2).

DNA extraction and amplification were successful in all the faecal samples chosen for analysis. After sequencing and bioinformatic process, with the Gillet primers, we recovered 214 ZOTUs: we ascribed 169 out of them to potential prey species, three to parasite species, 14 to environmental pollution DNA (e.g., fungi), and 89 could not be identified; ZOTUs assigned to *M. schreibersii* were amplified in all faecal samples. With the Zeale primers, we got 325 ZOTUs, of which 275 were ascribed to potential prey, and the rest were left unassigned. Combining Gillet and Zeale primers, we identified 187 different arthropods at the species level in bats' faeces, including the bat parasite *Nycteribia schmidtii* (Diptera, Hippoboscidae). We discarded 17 species out of them because they were previously recorded neither in the Iberian Peninsula nor within the 1000 km range in North Africa (Supplementary Material 5). Therefore, we classified 169 species as prey consumed by *M. schreibersii* (Supplementary Material 6).

Most species were consumed only once (71 spp) or twice (29 spp), and only 24 had frequency of occurrences (FOO) equal to or above 10%. Regarding species richness and wPOO, the most relevant prey groups at the order level were Lepidoptera, followed by Diptera, Hemiptera and Ephemeroptera (Table 2). Only 30 species showed wPOO ≥ 1% (Supplementary Material 6). They included 20 moth species (prevailing Noctuidae), six Diptera, three Ephemeroptera and one Hemiptera. Following wPOO, the most consumed species were the crambid moth *Thopeutis gallerielus*, followed by noctuids *Agrotis segetum*, *A. ipsilon* and *Leucania loreyi*, and the cranefly *Symplecta pilipes* (Diptera, Limoniidae), respectively. Besides, the most frequently consumed—in terms of FOO—were the noctuid moth *A. segetum*, followed by Ephemeroptera *Caenis luctuosa*, *Ephoron virgo* and *Chroroterpes picteti*, and the noctuid moths *L. loreyi*, *Autographa gamma* and *A. ipsilon*, respectively.

**Table 2:** Main groups of prey groups consumed by *M. schreibersii* throughout the studied period, showing the species richness (Sp.) and pooled weighted percentage of occurrences (wPOO%) of each group.

	Sp.	wPOO%
Lepidoptera	123	72.34
Diptera	29	14.97
Ephemeroptera	3	8.22
Hemiptera	4	3.88
Coleoptera	3	0.26
Orthoptera	1	0.18
Neuroptera	1	0.04
Blattodea	1	0.04
Trichoptera	1	0.03
Araneae	1	0.02

### Temporal variations in diet

The diet of *M. schreibersii* changed seasonally in terms of total consumed species richness, the average number of species per faecal sample, and the most consumed prey (Table 3). The diet richness was higher in spring, late summer, and autumn. The most frequently consumed prey species changed every sampling session, except for *T. galleriellus*, which showed the highest wPOO in three consecutive sets from 18 June to 16 July.

**Table 3:** Seasonal variation of consumed prey, showing for each two weeks the recorded species richness (Sp. rich), the average number of species (Av. sp.), it’s range (Range), the maximum weighted percentage of occurrence (Max wPOO*) and in which species (*sp.), and the maximum frequency of occurrence (Max FOO**) and in which species (**sp.): Aip, *Agrotis ipsilon*; Ase, *Agrotis segetum*; Aga, *Autographa gamma*; Clu, *Caenis luctuosa*; Evi, *Ephoron virgo*; Llo, *Leucania loreyi*; Npr, *Noctua pronuba*; Nsq, *Nola squalida*; Psa, *Peridroma saucia*; Pci, *Prays citri*; Spi, *Symplecta pilipes*; Tpi, *Thaumetopoea pityocampa*; Tga, *Thopeutis galleriellus.*

Date	sp. rich	Av. sp.	Range	Max wPOO*	*sp.	Max FOO**	**sp.
11.V	44	10	4–25	14.8	**Npr**	100	**Npr**
21.V	23	5.1	3–10	12.1	**Pci**	60	**Pci**
04.VI	35	5.5	3–12	19.6	Spi	50	Spi
18.VI	38	6.5	2–15	11.7	Tga	50	Ase
02.VII	16	3.4	2–7	31.4	Tga	90	Tga
16.VII	19	3.5	1–6	25.8	Tga	50	Tga
30.VII	36	4.6	1–17	25.8	Nsq	50	Nsq
13.VIII	38	8.7	3–16	9.6	Clu	70	Clu
27.VIII	52	10.9	5–20	10.3	**Tpi**	90	**Tpi**
10.IX	53	10.8	3–21	9.5	**Aga**	60	**Tpi**, **Aga**, Evi, Clu
24.IX	45	9.2	2–24	17.8	**Ase**	90	**Ase**
08.X	49	11.8	2–23	12.1	Llo	80	Llo, **Ase**
22.X	43	10.3	2–29	14.8	**Aip**	100	**Aip**
05.XI	28	7.8	2–22	10.0	Llo	60	Llo, **Psa**

Pest species are marked in bold.

**Figure 3:**
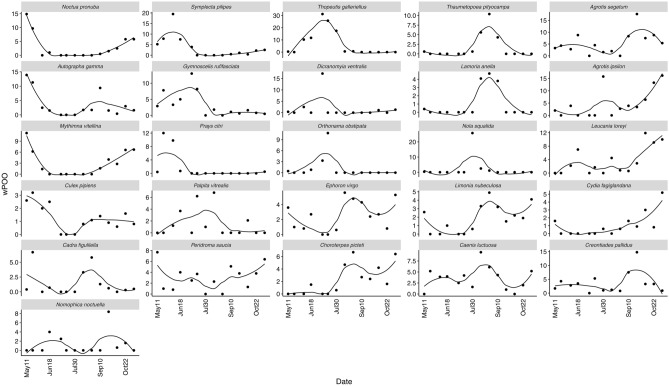
Observed wPOO values of 26 species selected from the canonical and uncanonical axes of the db-RDA: the points represent the observed wPOO values, and the trend is represented with LOESS smoother (span 0.7). Plots are ordered roughly according to the seasonality of the consumption peak (from left to right) and the range of wPOO values (from top to bottom).

To further study the temporality of the diet, we chose those with the highest and lowest scores in the significant axes (22 species in total) and the first two unconstrained axes (four additional species) of the db-RDA (Table 4, Supplementary Material 1). The consumption of all 26 species is portrayed in Fig. 3 using a LOESS smoother (span 0.7). The consumption of these species was variable throughout the year, and each one followed a different tendency. Seasonally, the most consumed prey in early May were the noctuids *Noctua pronuba* and *M. vitellina*, followed by the mayfly *E. virgo* and the mosquito *C. pipiens*. They are gradually substituted in late May and early June by the citric pest moth *P. citri*. In early summer, the geometrid *G. rufifasciata*, the crambid moth *T. galleriellus *and the nolid moth *N. squalida* sequentially replaced the cranefly *D. ventralis*. Throughout August, consumption of the mayflies *E. virgo* and *C. picteti* is overcome by solid predation upon the pine moth *T. pityocampa* (Notodontidae), leading to second consumption peaks of the noctuids *N. pronuba* and *M. vitellina* in autumn*.*

**Table 4:** List of species selected from the db-RDA.

Species	wPOO	Habitat	Source
*Thopeutis galleriellus*	7.0	Wet grasslands, marshes and forest-steppe biotopes along rivers	^6,12^
***Agrotis segetum***	5.4	Partly vegetated fields, embankments, patches in gardens, sand pits or gappy grasslands	^7^
***Agrotis ipsilon***	4.8	Cosmopolite	^7^
*Symplecta pilipes*	3.6	Associated with water or humid habitats	^2^
***Creontiades pallidus***	3.6	A variety of Mediterranean crops	^4,12^
*Leucania loreyi*	3.6	Grasslands and cultivated areas	^7,8^
*Caenis luctuosa*	3.5	Marshes, wetlands and rivers	^3^
***Autographa gamma***	3.5	Cosmopolite	^7^
*Gymnoscelis rufifasciata*	3.4	Warm and sunny places of all kinds	^7^
***Noctua pronuba***	3.0	Cosmopolite	^7^
***Peridroma saucia***	3.0	dry warm landscapes, extensive agricultural areas, garigues, ruderal terrain, moist habitats	^8^
*Mythimna vitellina*	2.9	Meadows, nutrient-poor grasslands, rural areas, roadsides and similar places	^7,8^
*Ephoron virgo*	2.5	Marshes, wetlands and rivers	^13^
*Choroterpes picteti*	2.2	Marshes, wetlands and rivers	^3^
*Nola squalida*	2.1	Wetlands and coastal areas	^12^
*Limonia nubeculosa*	1.8	Associated with water or humid habitats	^2^
***Prays citri***	1.7	Citrus plantations	^9^
*Dicranomyia ventralis*	1.6	Marshes, wetlands and rivers	^2^
***Palpita vitrealis***	1.5	Widespread in the Mediterranean region, larvae feed on olive, among others	^5^
***Thaumetopoea pityocampa***	1.5	Pine woodland	^8^
*Cadra figulilella*	1.4	Places where fruits and/or berries are present	^10^
***Culex pipiens***	1.2	Around any type of water body, where they breed	^1^
*Nomophila noctuella*	1.2	Grasslands and herbaceous rainfed crops	^5^
*Orthonama obstipata*	1.1	Cosmopolite	^7^
*Cydia fagiglandana*	1.0	Oak and beech woodland	^10^
*Lamoria anella*	0.9	Cosmopolite, larvae are associated with nests of *Vespula sylvestris* and *Polistes gallicus*	^11^

For each species the Weighted Percentage of Occurrence (wPOO) is given, as well as a brief description of their preferred habitat. The pest species and disease vectors are marked in bold. Sources in Supplementary Materials 2 and 4.

**Figure 4:**
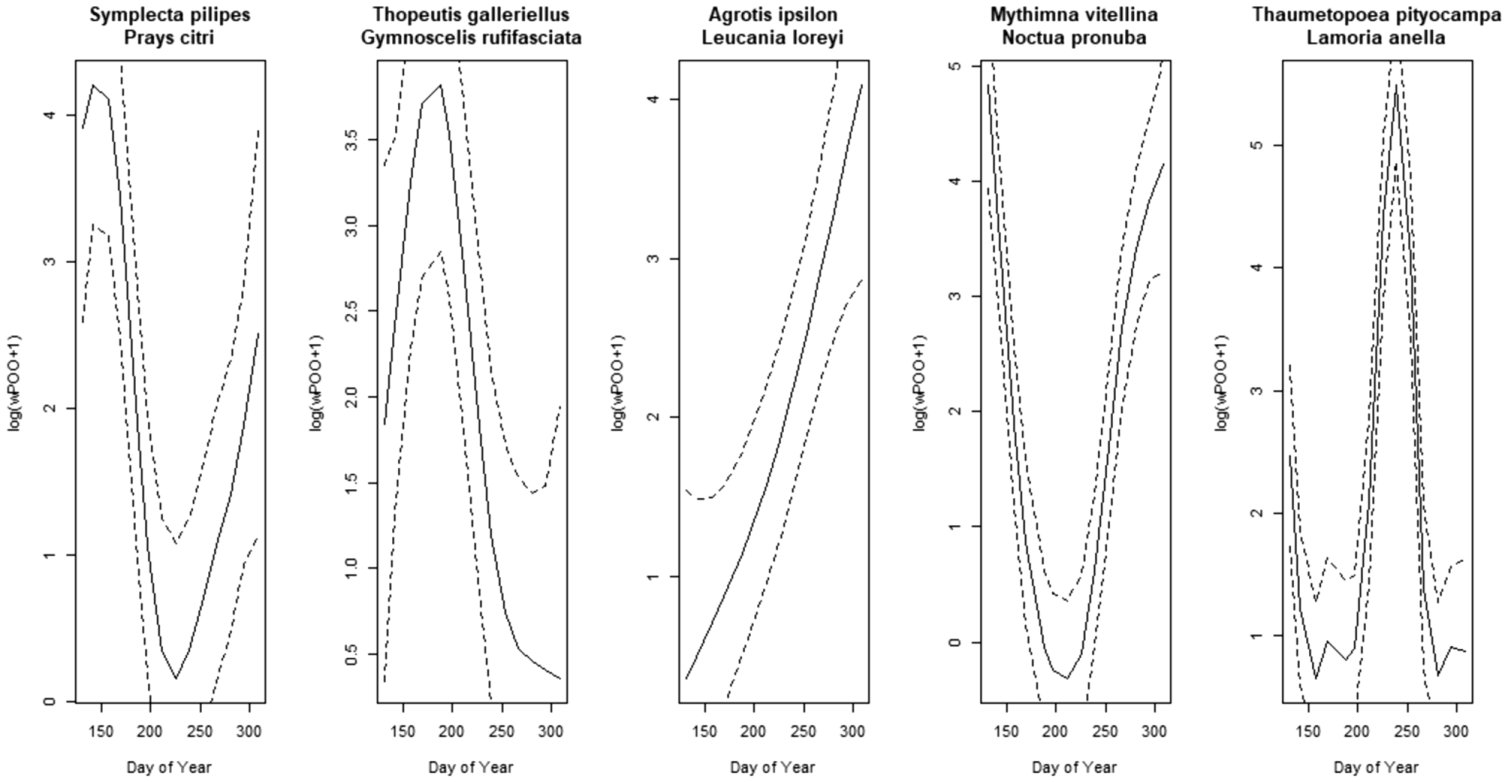
Smoothing curves (solid line) obtained by the GAMM model with statistical significance at the 1% level. Dashed lines represent 95% confidence bands.

The 22 species influential in the canonical axes of the db-RDA were grouped and modelled against date as the continuous factor day-of-the-year in a Generalized Additive Mixed Model (Eq. 1, in Material and Methods). Five of the eleven smoothers were significant at the 0.01 value: *A. ipsilon* + *L. loreyi, M. vitellina* + *N. pronuba*, *S. pilipes* + *P. citri*, *T. pityocampa* + *L. anella* and *T. galleriellus* + *G. rufifasciata* (Table 5). These species show the highest variability in their consumption throughout the sampling season, being only identified in a particular sampling period. The estimated wPOO values of the five significant smoothers (Fig. 4) confirm the tendencies portrayed in Fig. 3. There is substantial turnover in the prey species consumed by *M. schreibersii*. The remaining species in the GAMM are still essential food items for our colony, even though we could not model a significant consumption pattern against time. Species like *A. segetum*, *Peridroma saucia* or *Palpita vitrealis* are consumed in great numbers throughout the year, providing a more constant food source for *M. schreibersii*, albeit without overshadowing more punctual, ephemeral resources like the ones mentioned above.

**Table 5:** Summary of the results of the GAMM model presented in Eq. (1).

Parametric coefficients					
	Estimate	Std. error	t-value	Pr( >|t|)	Signif.
(Intercept)	1.9231	0.2094	9.184	< 2e-16	***

Approximate significance of smooth terms					
	edf	F	p-value	Signif.	
*Agrotis ipsilon* *Leucania loreyi*	1.517	8.863	0.000580	**	
*Autographa gamma* *Peridroma saucia*	2.781	2.700	0.029290	*	
*Caenis luctuosa* *Cadra figulilella* *Choroterpes picteti*	1.001	3.321	0.069554		
*Creontiades pallidus* *Nomophila noctuella* *Agrotis segetum*	1.001	0.949	0.330427		
*Dicranomyia ventralis*	1.001	0.090	0.765675		
*Nola squalida* *Orthonama obstipata*	2.151	1.924	0.121142		
*Mythimna vitellina* *Noctua pronuba*	4.878	34.544	< 2e−16	**	
*Palpita vitreallis*	1.885	0.810	0.366543		
*Symplecte pilipes* *Prays citri*	4.351	10.074	2.77e−07	**	
*Thaumetopoea pityocampa* *Lamoria anella*	8.078	34.991	< 2e−16	**	
*Thopeutis galleriellus* *Gymnoscelis rufifasciata*	3.550	7.077	0.000126	*	
R-sq. (adj) 0.529					

Significance codes: 0 ‘***’ 0.001 ‘**’ 0.01 ‘*’ 0.05 ‘.’ 0.1 ‘ ’ 1.

### Landscape requirements of the main prey

**Figure 5:**
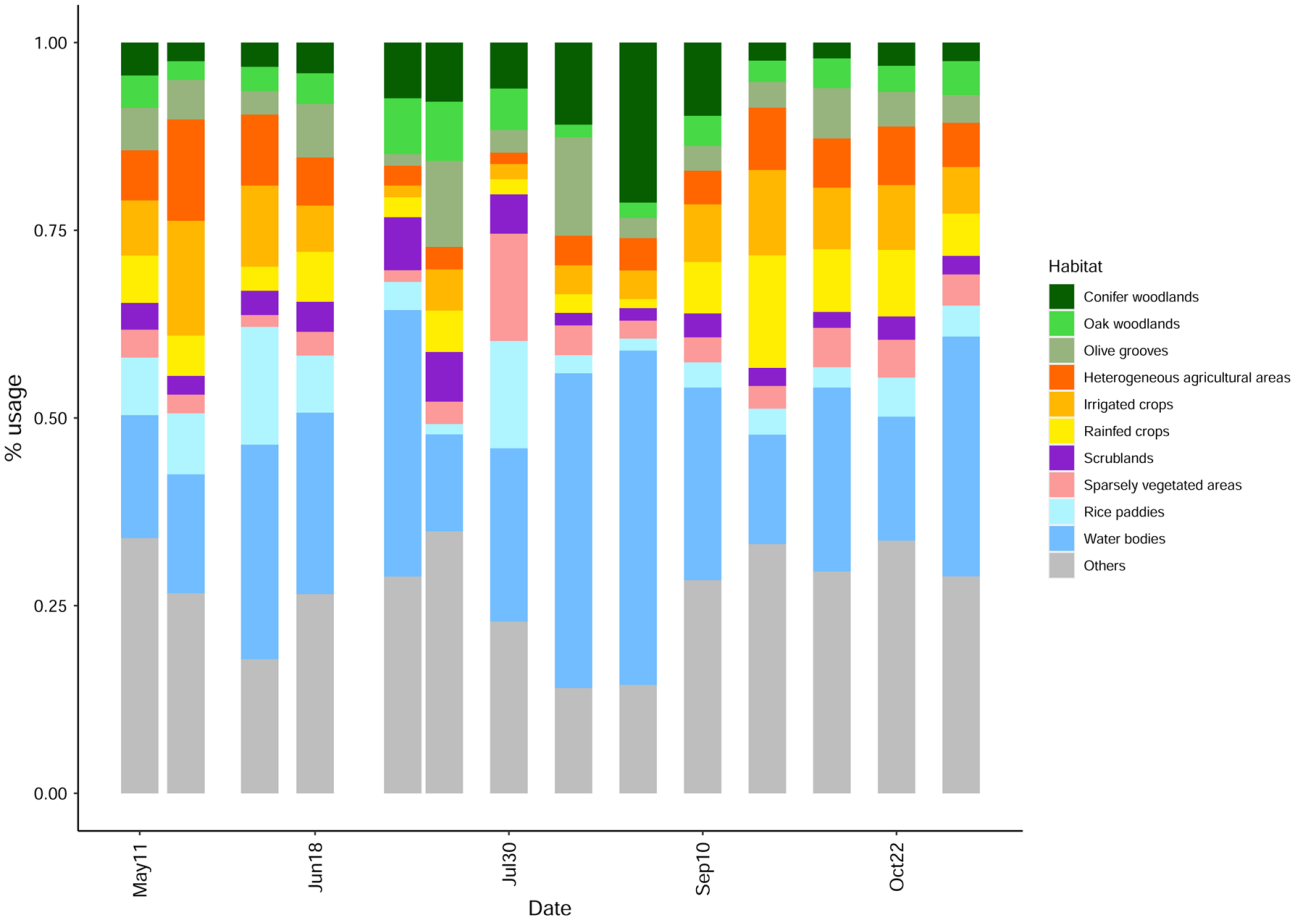
Percent of use of different available habitat types by *Miniopterus schreibersii* based on the wPOO of the captured prey and their source habitats.

Assessing the environmental requirements of *M. schreibersii* based on their prey’s source habitats, we must conclude that they depend on very diverse habitat types, from water bodies and rice paddies to sparsely vegetated areas and scrublands, different types of cropland, and diverse woodlands as well. Moreover, the weight of those habitats in their most consumed prey varies seasonally (Fig. 5).

**Figure 6:**
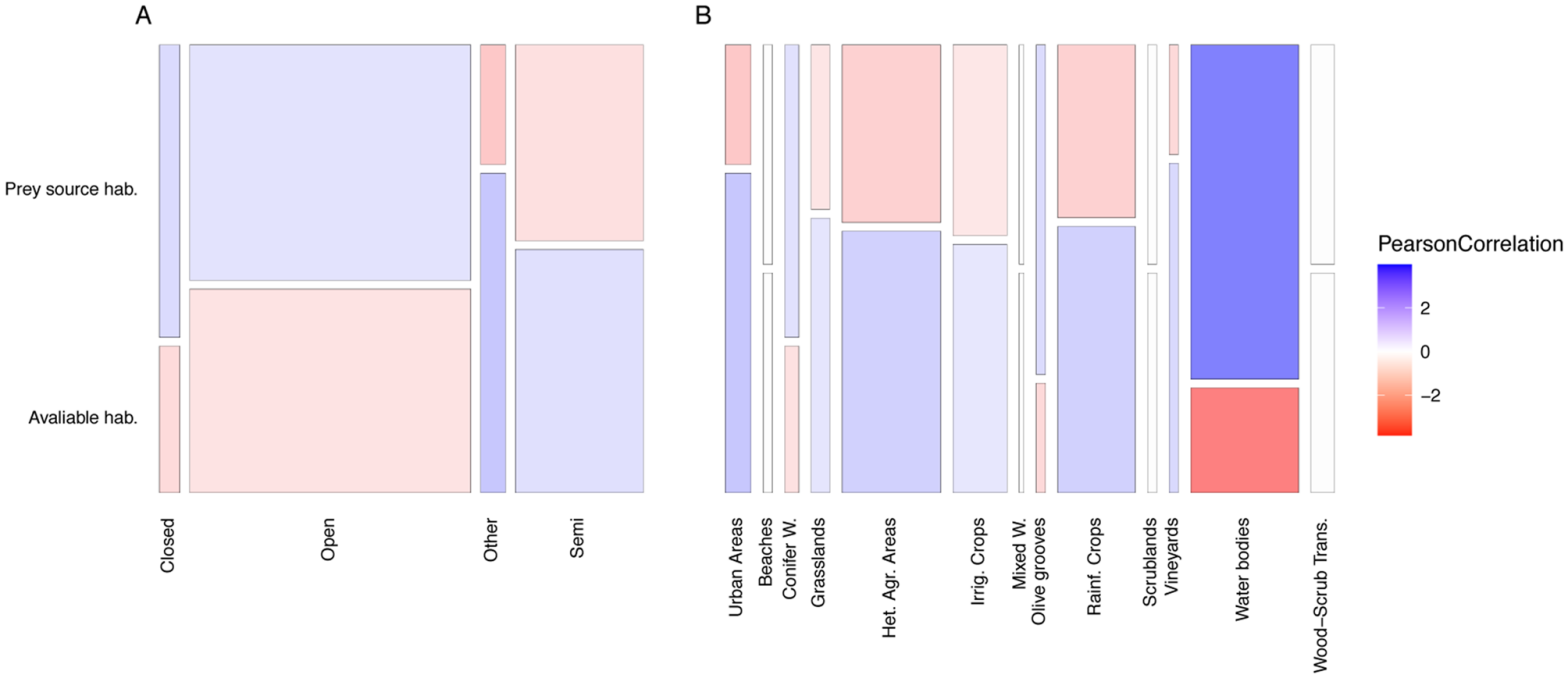
Mosaic plots of available habitat types versus source habitats of the most consumed prey species. Habitat types are classified according to (**A**) the vegetation density and (**B**) the vegetation type. The bars’ width represents the combined percentage of that category in the dataset, and colours show Pearson’s correlation coefficients.

The mosaic plot in Fig. 6 portrays the differences between available and prey’s source habitats. The Chi-square test of independence showed no difference between the available and prey's source habitat types (Fig. 6A) according to the vegetation density (X-squared = 4.5692, df = NA, p-value = 0.2054). For the vegetation coverage (Fig. 6B), significant differences were found (X-squared = 21.357, df = NA, p-value = 0.03448), albeit not very pronounced. Pearson’s residuals showed that "Water Bodies" contributed to 28.8% of the differences in the Chi-square statistic. This category was the only one significantly different, being more important as prey source by *M. schreibersii* than expected from its availability around the colony. This suggests that the colony studied frequently consumed prey associated with water bodies such as *C. luctuosa*, *E. virgo*, *C. pipiens* or *L. nubeculosa,* which could be hunted close to drinking spots. No other vegetation type shows a significant difference between the two datasets, indicating that *M. schreibersii* exploits prey of all habitats equally around the colony.

### Pest consumption

**Figure 7:**
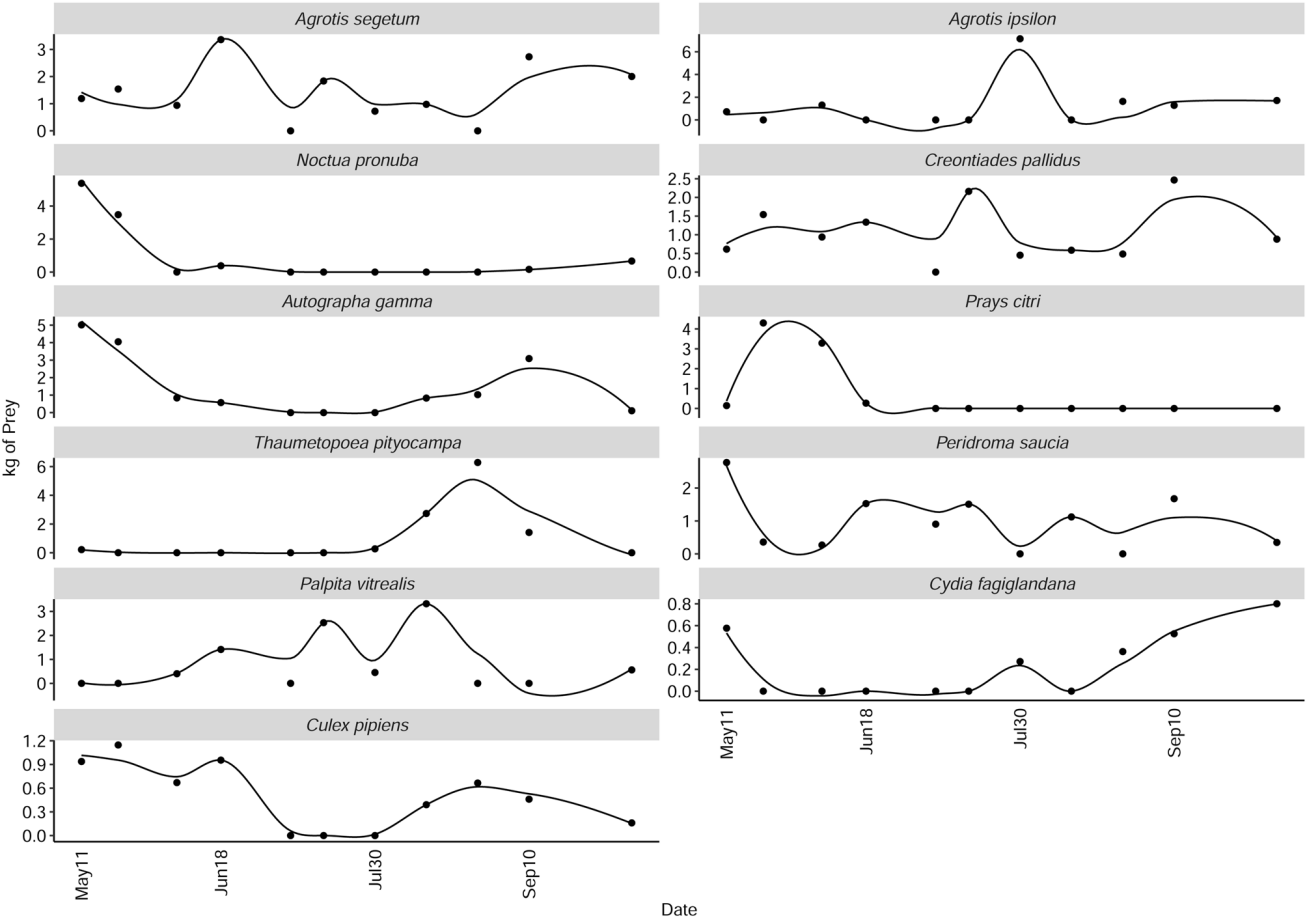
Estimated quantities of pest species and disease vectors consumed, in kilograms, throughout the sampling season. The dots represent the estimations derived from the observed wPOO. The line represents a LOESS smoother (span 0.5).

Notably, 39 species identified as consumed are categorized as a pest in different degrees. Consulting the results of the db-RDA and the GAMM models, we identified ten pest species consumed in significant numbers by this colony of *M. schreibersii*. We calculated their overall consumption based on wPOO for that specific prey and the colony size, resulting in different consumption patterns for each pest species (Fig. 7). Estimation was possible from the 11th of May to the 10th of October 2018, when we monitored the colony size.

Quantitatively, the consumption of agricultural pest insects by bats summed up to 1610.3 kg in 5 months, of which 1467.3 kg correspond to ten species. The most consumed was *A. segetum*—234 kg—with a steady consumption year-round and higher peaks in mid-June and autumn (Fig. 7). The following most consumed pests were *A. gamma* (195 kg), *C. pallidus* (186 kg), *T. pityocampa* (173 kg) and *A. ipsilon* (163 kg). The consumption of *C. pallidus* was relatively uniform. Contrarily, predation upon *T. pityocampa* was limited to its flying season, starting in August: in a single day, the colony consumed up to 6 kg of *T. pityocampa*. With lower numbers, *P. saucia* (142 kg), *P. vitrealis* (141 kg) and *P. citri* (109 kg) were also heavily consumed. While the bats preyed upon the first two throughout the sampling season, *P. citri* was only eaten during its flying season in spring. *N. pronuba* (86 kg) was only consumed in early May, and *C. fagiglandana* (32 kg) primarily in the late season. Finally, *C. pipiens* (73 kg) was consumed mostly during May and August.

Assessed by weighting the total consumption with the surface of prey-habitat surface in the study area, the highest predation intensity occurs upon the more habitat-specialist pest species:

- *P. vitrealis*, 4.7 kg/100 ha in olive groves.
- *T. pityocampa,* 1.73 kg/100 ha in conifer plantations.
- *C. fagiglandana,* 0.53 kg/100 ha in *Quercus* woodland.
- *C. pallidus,* 0.37 kg/100 ha in irrigated crops.

On the contrary, the pests listed above with the highest consumption numbers inhabit varying habitat types, and consequently, their predation intensity will be relatively lower.

## Discussion

In this study, we combined the population monitoring of a colony of *M. schreibersii* with an intensive sampling and thorough metabarcoding analysis of the faecal samples to show that: (1) the bats’ diet continuously varies, showing a succession of peak prey, which includes both pest and non-pest insects; (2) many of those most-consumed-species have different habitat preferences/dependences (Fig. 5), bats positively selecting water bodies but somehow using—or taking benefit from—all of the other habitat types as well; (3) bats’ predation upon pest insects is quantitatively high; and (4) their suppression effect may be relevant, mainly in patchy heterogeneous landscapes, where foraging bats may concentrate in successive outbursts of pests, affecting to different crops or woodlands.

### Diet diversity and variation

Our results fit well with *M. schreibersii* being a fast-flying, open-space-forager^61,84^ and aerial-hawking bat [e.g.,^85,86^], specialised in fluttering flying insects, such as moths, dipterans, and others. Our analysis confirms moth —mostly Noctuidae, but also Crambidae, Geometridae, Pyralidae, Notodontidae and others—constitute their staple diet, in agreement with both the morphological^87,88^ and molecular studies^26^ hitherto published.

Among the most consumed prey, the noctuid moths *A. gamma, A. segetum,* *A. ipsilon, N. pronuba, P. saucia, M. vitellina* or *Nomophila noctuella* are widespread and abundant insects, which seasonally constitute the staple food of many moth-specialist bats: namely *Plecotus *sp.^18,48^; *Rhinolophus euryale*^89^; *Tadarida teniotis*^47^; and also *M. schreibersii*^26^. The notodontid pine moth *T. pityocampa* is a frequent prey of several moth-specialist bats as well, including not only forest bats but also *M. schreibersii* and other open-space-foragers^28^. Similarly, the main Diptera consumed are common prey of insectivorous bats: e.g., *L. nubeculosa* is consumed by *R. hipposideros* and *R. ferrumequinum*^70,90^, *T. teniotis*^47^, *Myotis daubentonii*^23,76^, *Myotis dasycneme*^23^, and *Plecotus auritus*^48^; *S. pilipes* by *M. daubentonii*^90^ and *R. hipposideros*^27^; and *D. ventralis* by *R. hipposideros*^27^ and *M. daubentonii*^90^.

Noteworthy, some of the most frequent prey in this study were seldom or never reported as bats' prey. Among these, the most frequently consumed species in this study, *T. galleriellus*^91^, is a crambid moth only known from very few distribution data in southern Europe (including the nearby Doñana National Park), the Middle East and Asia^92,93^. Like many other crambid moths, it seems linked chiefly to wet grassland and marshes. Similarly, the nolid moth *N. squalida* and *C. pallidus*, a hemipteran pest from the family Miridae, had not been recorded as bats prey before. These findings indicate that new research on insectivorous bats' diets in different localities and seasons will unveil other relevant prey species, helping complete our knowledge of the trophic webs^35^.

Also notable is the intense consumption of Ephemeroptera, only punctually reported as prey for this bat^26,88^. In our study, mayflies *C. luctuosa, E. virgo* and *C. picteti* were relatively important prey, showing several peaks throughout the year (Fig. 4), in accordance with *M. schreibersii* foraging most actively over rivers and wetlands^94,95^. In contrast, we detected neither Trichoptera nor Chrysopidae (Neuroptera), previously reported as prey^26,87,88^.

Insectivorous bats are selective opportunistic predators that actively search areas with abundant prey sources^30,96–99^, adjusting their diet to prey abundance. Slight differences in *M. schreibersii* diet composition have been described among localities and seasonally. The metabarcoding analysis of *M. schreibersii'*s faeces across Europe showed their diet varied among localities linked to habitat, indicating that the dietary diversity was negatively related to the area of intensive agricultural fields^26^. Moreover, by morphological identification of prey remains in faeces at the order and family levels, a study carried out in a single roost in Slovenia throughout the year showed *M. schreibersii* as a moth specialist that opportunistically switches among other available prey, with a more diverse diet in spring and autumn^87^.

With a detailed identification of prey at the species level, our study shows *M. schreibersii* hunting more opportunistically in spring and from late summer onwards but more selectively in summer. This pattern is consistent with the behaviour described for several insectivorous bats: e.g., species of genus *Plecotus*^18^, *Pipistrellus kuhlii*^100^, or *R. ferrumequinum*^101^.

Moreover, according to our first hypothesis, our results confirm *M. schreibersii* gradually shifts from one peak-prey species to another, most likely tracking varying insect availability. Many of those prey species show one single consumption peak throughout the studied period (e.g., *T. galleriellus, S. pilipes*, *T. pityocampa* or *C. pallidus*, in Fig. 4). Others show two maxima, likely answering to availability. Among the later, some are migratory species, more abundant in spring and autumn (e.g., *N. pronuba, A. gamma* or *A. ipsilon*). Others produce two or more broods throughout the year (e.g., *A. gamma, A. segetum,* *N. noctuella*)^102–105^.

### Habitat requirements

Supporting our second hypothesis, many of the most consumed *M. schreibersii* prey depend on different landscapes or habitats, which implies seasonal changes in the required environmental resources for the bats (Fig. 5). While some prey species may inhabit varying habitats and crops (e.g., *A. gamma, Agrotis sp., Spodoptera sp*.), others are more clearly linked to specific landscape elements: e.g., Ephemeroptera to water bodies; limoniid and psychodid Diptera to wetlands and damp spots; *T. pityocampa* to conifer woodland; *P. citri* to citrus plantations; *C. flagigandana* to *Quercus* and beech forests; *T. galleriellus* to wet grasslands and marshes, et cetera (see Table 4).

In insectivorous bats, habitat selection is a hierarchical decision-making process^49^: the species' morpho-ecological specializations drive it first, and subsequently, factors linked to optimal foraging prevail, e.g., prey availability^49,99,100,106^, distance to the roost^107^, landscape features^101^, or interspecific competition^108,109^.

For high-flying, open-space foragers like *M. schreibersii*, the importance of the habitat types underlying their hunting grounds is challenging to ascertain beyond the prey availability above them (e.g., *Vespertilio murinus*^110,111^; *Eptesicus nilssonii*^112^; *Nyctalus lasiopterus*^113^; *Hypsugo savii*^114^. Radiotracking studies on *M. schreibersii* in France have shown high habitat flexibility: they mostly forage over urban areas, followed by open spaces, woodlands, orchards and parks^62,115^; meanwhile, they use water bodies less frequently than expected from availability. On the contrary, in Italy, *M. schreibersii* forage in riverine forests^94^. They mainly use open areas and water bodies, followed by lighted urban areas, oak forests, and water lines in Portugal^95,107^. This foraging plasticity fits well with the habitat diversity of the principal prey in the present study (Table 4). Moreover, it also mirrors the main habitats available in the colony's foraging range (Fig. 1) and is consistent with our data for the whole active season, revealing no habitat selection (Fig. 6).

Our data do not reveal whether the bats foraged in any specific habitat but link each prey species to some of them. In fact, bats may often hunt prey originating in habitats different from where they are hunted^19^. Thus, bats' foraging requirements are not constrained to their foraging habitats, but also encompass their prey requirements^116–118^. Consequently, in our case study, *M. schreibersii* will seasonally rely on the diverse habitat around their roost, if not to directly forage therein, at least as sources of the prey they mostly hunt.

Several pieces of research showed the foraging activity of insectivorous bats in agroecosystems is linked to habitat heterogeneity [e.g.,^98,119–121^]. This correlation has been explained because farmland heterogeneity is a crucial predictor of overall biodiversity^122–124^ and of bats’ prey insects in particular^98,125,126^. Therefore, landscape heterogeneity increases spatial niche partitioning and thereby reduces the effects of interspecific competition among bat species^127^. Beyond that, our results indicate that habitat heterogeneity in agroecosystems is of great value in itself, also for single bat species like *M. schreibersii,* allowing them to shift from one insect outburst to the next, sequentially occurring in different habitat types. Moreover, they point out that aerospace is the true foraging habitat for high-flying, open-space forager bats. The underlying habitat types may be seasonally relevant either as source of prey outbursts—e.g., crops, forest, water bodies or other habitats—, as foreseeable prey attractors —streetlights or waterbodies—, or because they provide linear landscape elements or landmarks helpful in commuting or foraging.

### Ecosystem services: which ones and how much?

Based on their extensive diet study across Europe, Aizpurua et al.^26^ pointed out that *M. schreibersii* might be a valuable asset as a pest suppressor. Our results confirm their forecast, as several agroforestry pests—and two disease vectors—are among the most consumed prey by this bat. Thus, among the 39 consumed species clategorized as pests in the EPPO Global Database^82^, in Spain 22 are major pests of several crops and woodland, and 18 are minor pests (Supplementary Material 4).

Moreover, *M. schreibersii* also preyed upon the hemipteran *Neophilaenus campestris,* which, if not directly regarded as a crop pest, is a potential vector of *Xylella fastidiosa*, a bacterium causing significant economic losses, especially in olive crops^128,129^. Noteworthy, we also recorded the nematoceran *C. pipiens* within the most consumed prey (Table 4), reaching 3.25% wPOO in mid-May. This mosquito is a vector of the Nile Fever disease, with several cases detected in the area in 2020, including one deceased^130^.

The insectivorous bats' diet metabarcoding studies have confirmed that these mammals prey upon many pest species [e.g.,^26–30,33,131^]. Nevertheless, deciphering to which extent bats consume them within intensive agroecosystems is crucial to talk about pest regulation or effectively assess the actual value of bat insectivory as an ecosystem service^35^. Even more to manage the negative impacts of pests through consumption by insectivorous bats^27^.

Quantitative assessment of diet composition is always challenging, implying several assumptions^132^. With the coming of metabarcoding techniques, additional difficulties appeared in turning the genomic analysis output into reliable quantitative diet data [e.g.,^30,78,133–136^]. Therefore, the assumptions underlying the quantitative analysis presented herein are debatable since they may either overestimate or underestimate specific consumptions. On the first hand, bats' total amount of insects consumed each night is open to discussion, ranging from 30 to 100% of their body weight [e.g.,^1,2,4^]. Therefore, our choice of 70% (following 1) might lead to overestimation, mostly because our sampling did not only happen during the breeding season and a high percentage of the samples belonged to males. Moreover, we did not consider that, due to meteorological factors, bats do not hunt in all nights—even if the climate is highly benign in the study area. On the other hand, the wPOO used to measure the importance of each prey in the diet tends to sub-estimate the largest or most abundantly consumed items^78,83^.

All in all, our quantitative approach determines the order of magnitude of the suppression a colony this size exerts on pests and disease vector insects in its foraging range. Besides, they will similarly affect the quantitative assessment of most main prey consumption. The exception will be the mosquitoes *Culex pipiens*, for wPOO may estimate the same consumed proportion, the bats having eaten anything from one single individual to hundreds of mosquitoes—so, we excluded them from the further discussion.

Notwithstanding the mentioned limitations, our data show that *M. schreibersii* heavily preys upon many pests. The generalist noctuid moths *A. segetum, A. gamma* and *A. ipsilon* quantitatively prevail. All are major pests of several horticultural crops and *Zea mays*, and minor of many others. It is also worth mentioning the high consumption of *C. pallidus*, a common pest in cotton lands and Solanaceae in the study area (Table 4; Supplementary Material 4).

To evaluate the ecosystem services bats provide consuming these pest insects, we must go beyond the absolute consumption numbers and consider the extension of croplands and woodland they affect in the colony's foraging range. Taking that into account, it is noteworthy that in our study the highest predation intensity happened upon woodland pests such as *P. vitrealis* (pest of olive groves), *T. pityocampa* (pest of conifers), or *C. fagiglandana* (pest of *Quercus* forests). Obviously, forestry pests such as *C. fagiglandana* are common prey of the forest bats *B. barbastellus* or *P. auritus*^137^. At first glance, the vigorous predation intensity on woodland pests observed in our study by an open space forager as *M. schreibersii* is more surprising. It indicates that these bats readily answer to the hot spots caused by the pest outbreaks, even in forests and other wooded areas, heavily hunting them, maybe above the canopy, along the forest edges, in clearings, or during insects commuting among patches. This intense predation upon forest pests is more noticeable considering that some of the host habitat patches likely exploited are far away from their roost—e.g., pine plantations for *T. pityocampa,* 28–30 km far. Others, such as the *Quercus* forests inhabited by *C. flagigandana*, are lacking in the 40 km around the roost, and we cannot discard that those bats fly further to profit from them (Fig. 1). The frequent consumption of forest pests by *M. schreibersii* and other open space foraging bats had been previously reported, e.g., consuming the pine moth *T. pityocampa*^28^ or outbreaks of the Asian gipsy moths (*Lymantria dispar*) in holm-oak woodlands^62^.

Our quantitative data suggest that such predation may be strong enough to restrain or even reduce pest damage, mainly when it affects patches of limited size. Moreover, the swift shift of their primary prey —and their habitats— suggests that *M. schreibersii* track pest abundance, increasing foraging activity when pests become more numerous and consequently decrease pest numbers, a requirement to exert control over pest populations^35^.

Accordingly, the bat population peak we observed in late August does not correspond to the incorporation of yearling individuals, who most likely began their foraging flights in early July (Fig. 2). Otherwise, this short-lasting rise might be due to the joining of bats from other roosts outside the study area, attracted to outbursts of prey such as *T. pytiocampa* and *L. anella*, whose consumption peaks are entirely coincidental in time.

This link between the spatial and social ecology and the availability of trophic resources was already foreseen by Rodrigues and Palmeirim^60^, who suggested that at least part of the seasonal displacements among roosts by *M. schreibersii* would answer to differential prey availability. Consequently, the tracking of pests and other outburst of insects by *M. schreibersii* would not only happen at the colony level but at a larger territorial scale, involving other nearby colonies of the metapopulation.

## Conclusions

This study confirms that *M. schreibersii* are selective opportunist predators of moths and other fluttering insects in the aerospace, shifting their diet to temporary peaks of prey availability in their foraging range. Throughout the year, they consume insects linked to diverse open habitats, including wetlands, grassland, and diverse croplands, but woodland as well. The importance of each prey habitat varies seasonally, depending on the insect phenology therein, making bats indirectly dependent on a diverse landscape for their primary prey source. Besides, *M. schreibersii* consumes high numbers of several agroforestry pests and at least two disease vector insects, confirming the value of this bat as a suppressor and potential regulator of detrimental insect populations. Thus, our results stress that it is crucial to preserve the funcionality of bats or other generalist insectivores, keeping the environmental conditions they require to thrive, particularly a heterogeneous landscape within the colony’s foraging area. Moreover, farmers and land managers should look for combining different crops and land patches whose major insect assemblies would present non-overlapping outbursts, based on their expected phenology.

### Supplementary Information

Supplementary Information 1.

Supplementary Information 2.

Supplementary Information 3.

Supplementary Information 4.

Supplementary Information 5.

Supplementary Information 6.

## Data Availability

The dataset generated during and/or analysed during the current study are available from the corresponding author on reasonable request.
