# Supplementary material for: Aerospace-foraging bats eat seasonably across varying habitats
Source: Sci Rep. 2023 Nov 10;13:19576. doi: 10.1038/s41598-023-46939-7 (PMC10638376; doi:10.1038/s41598-023-46939-7)
Supplement: Supplementary file 1 — Supplementary Information 1. [file 41598_2023_46939_MOESM1_ESM.pdf]

Title: Aerospace-foraging bats eat seasonably across varying habitats: implications for ecosystem services.

Authors: Joxerra AIHARTZA[1], Nerea VALLEJO[1], Miren ALDASORO[1], Juan L GARCIA-MUDARRA[2], Urtzi GOITI[1], Jesus NOGUERAS[2], Carlos IBAÑEZ[2]

Affiliations:

[1] Dpt. of Zoology and Animal cell Biology, University of the Basque Country UPV/EHU. Sarriena s/n, E48940, Leioa, The Basque Country.

[2] Estación Biológica de Doñana (CSIC), P.O. Box 1056, E41080, Sevilla, Spain.

Corresponding author: Joxerra Aihartza, joxerra.aihartza@ehu.eus;

Supplementary Material 1:

Distance based redundancy analysis for the identification of seasonally significant prey items

Methodology

We used distance-based redundancy analysis (db-RDA: Legendre and Anderson 1999) to study the multivariate relationship of the diet with each of the sampling dates. Unlike regular RDA, db-RDA uses a dissimilarity matrix of the desired metric in a Principal Coordinate Analysis, which yields a set of principal coordinates usable in regular RDA. Db-RDA allows dissimilarity metrics appropriate for community data not applicable in regular RDA. We removed those species present in less than five samples in our data and based our RDA on a Bray Curtis dissimilarity matrix of the resulting dataset. The db-RDA was performed using non-scaled data (Covariance Matrix RDA), setting the sampling date as an explanatory variable in the function *capscale* of package *vegan* (Oksanen et al. 2020). The function *anova.cca* was used to test the significance of the linear relationship between the sampling date and the db-RDA and the significance of constrained axes, that is, the axes explained by the sampling date. We then selected the prey species with the highest and lowest coefficients in each significant constrained axis. From a temporal perspective, these species will be the most influential and have the highest wPOO values on each sampling date. Due to the high variability of the diet, the first two unconstrained axes had bigger eigenvalues than the second constrained axis, suggesting a pattern in our results that could not be explained by the explanatory variable (sampling date) alone. Therefore, we selected prey species for these two axes the same way we did for the constrained axes.

Interpretation of the db-RDA

Sampling-date significantly affected the db-RDA (p-value = 0.001); it alone explains 27% of the variation in the diet. The first six axes of the db-RDA were significant, challenging their graphical interpretation.

However, most constrained variation (65%) and total variation (17%) are retained within the first three axes. In the db-RDA triplots, samples from the same day or close dates —grouped by colours—appear overall grouped (Figs. S1a, S1b and S1c; the distance is not directly interpretable in this scaling). The “consequential” species with higher absolute coefficient values appear further away from the centre in the RDA triplot, pointed out with specific legends.

Supplementary Fig. S1 db-RDA plots showing the constrained axes 1, 2 and 3 (plots a to c), which represent 65% of the constrained variation and 17% of the total variation, and unconstrained axes 1 and 2 (plot d), which represent 14% of the total variation: CadFig, *Cadra figulilella*; NocPro, *Noctua pronuba*; AgrIps, *Agrotis ipsilon*; NomNoc, *Nomophila noctulella*; DicVen, *Dicranomia ventralis*; LanAne, *Lamoria anella*; PerSau, *Peridroma saucia*; AgrSeq, *Agrotis segetum*; PalVit, *Pales pavida*; SymPil, *Symplecta pilipes*; NolSqu, *Nolia squalida*; AutGam, *Autographa gamma*; ThoGal, *Thopeutis galleriellus*; CaeLuc, *Caenis luctuosa*; ThaPit, *Thaumetopoea pityocampa*; LeuLor, *Leucania loreyi*; GymRuf, *Gymnoscelis rufifasciata*; ChoPic, *Choroterpes picteti*; PraCit, *Prays citri*; MytVit, *Mytimna vitellina*; OrtObs, *Orthonama obstipata*; CrePal, *Creontiades pallidus*; EphVir, *Ephoron virgo*; LimNub, *Limonia nubeculosa*; CydFag, *Cydia fagiglandana*; CulPip*, Culex pipiens*

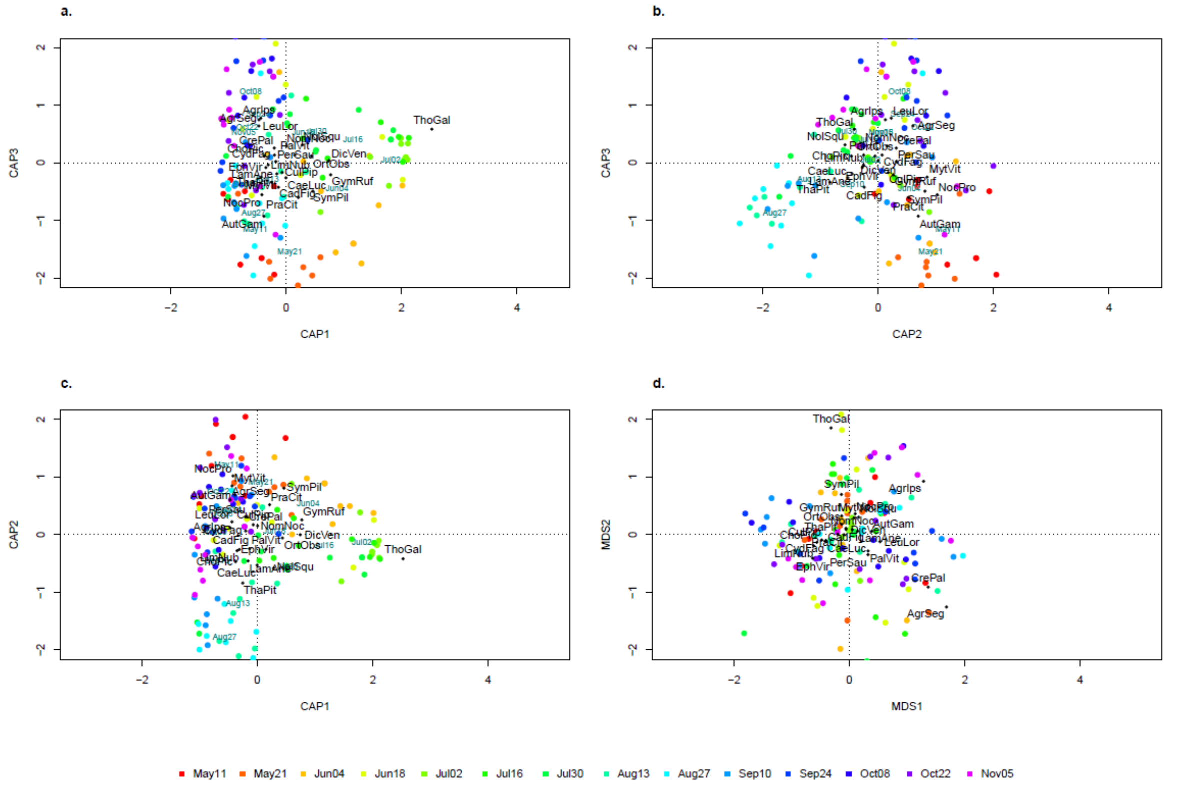

The first axis (CAP1-axis in Figs. S1a and S1c) separates some species with positive scores, related to samples from June and July (coloured yellow/green, on the right), from the others (with negative scores). Among the most consumed prey species, *T. galleriellus* dominates in this period, followed by *Dicranomyia*

ventralis, Gymnoscelis rufifasciata or *S. pilipes,* scoring lower in the axis. On the contrary (with negative scores), e.g., *A. ipsilon, A. segetum* or *Mythimna vitellina* show opposite trends, not being consumed in June.

The second axis (CAP2-axis in Figs. S1b and S1c) separates those samples collected in August (light blue in the plots) from the rest. The two species, *Thaumetopoea pityocampa* and *Lamoria anella,* are correlated with them, while again, *A. ipsilon*, *A. segetum* and *M. vitellina* appear on the opposite side of the graph.

The third axis (CAP3-axis in Figs. S1a and S1b) separates the samples taken in May (marked red and orange, where *A. gamma* and *Prays citri* dominate) from those from October and November (dark blue and purple in the graph, correlated to *A. ipsilon, L. loreyi* and *A. segetum*).

The overall variability explained by the db-RDA, especially the first two axes described above, is relatively low. This is not surprising, as the number of species in the analysis is high, and most of them are only consumed by one or two bat individuals. Besides, the first two unconstrained axes (MDS1 and MDS2 in Fig. 3.d) —unrelated to the explanatory variable sampling date—are relatively important, as their eigenvalues are higher than the second constrained axis, and they alone explain almost as much variation as the first three canonical axes (14%). As expected, the ordination of samples shows no clear pattern regarding the sampling date. Species important in these two axes include some mentioned above, like *T. galleriellus*, and new ones, like *E. virgo*, *Limonia nubeculosa*, *Cydia fagiglandana* or *Culex pipiens,* which score high in the first non-canonical axis.

Bibliography

Legendre P, Anderson M (1999) Distance-based redundancy analysis: Testing multispecies responses in multifactorial ecological experiments. Ecological Monography 69: 1-24. https://doi.org/10.1890/0012-9615(1999)069[0001:DBRATM]2.0.CO;2

Oksanen J, Blanchet FG, Friendly M, Kindt R, Legendre P, McGlinn D, Minchin PR, O'Hara RB, Simpson GL, Solymos P, Stevens MHH, Szoecs E, Wagner H (2020) vegan: Community Ecology Package. R package version 2.5-7. https://CRAN.R-project.org/package=vegan
