# Supplementary material for: Aerospace-foraging bats eat seasonably across varying habitats
Source: Sci Rep. 2023 Nov 10;13:19576. doi: 10.1038/s41598-023-46939-7 (PMC10638376; doi:10.1038/s41598-023-46939-7)
Supplement: Supplementary file 2 — Supplementary Information 2. [file 41598_2023_46939_MOESM2_ESM.pdf]

Title: Aerospace-foraging bats eat seasonably across varying habitats: implications for ecosystem services.

Authors: Joxerra AIHARTZA[1], Nerea VALLEJO[1], Miren ALDASORO[1], Juan L GARCIA-MUDARRA[2], Urtzi GOITI[1], Jesus NOGUERAS[2], Carlos IBAÑEZ[2]

Affiliations:

[1] Dpt. of Zoology and Animal cell Biology, University of the Basque Country UPV/EHU. Sarriena s/n, E48940, Leioa, The Basque Country.

[2] Estación Biológica de Doñana (CSIC), P.O. Box 1056, E41080, Sevilla, Spain.

Corresponding author: Joxerra Aihartza, joxerra.aihartza@ehu.eus;

Supplementary Material 2:

References of sources cited in Table IV (in the main text), regarding the habitats of the most consumed prey species.

[1] www.ecdc.europa.eu
[2] ccw.naturalis.nl
[3] Gallardo-Mayenco, A. (1994). Freshwater macroinvertebrate distribution in two bains with different salinity gradients (Guadalete and Guadaira river basins, south-western Spain)- *International Journal of Salt Lake Research*, 3, 75-91.
[4] Salvador-Sola, S. (2015). Creontiades pallidus. Cajamar, Fichas de transferencia. https://www.cajamar.es/storage/documents/008-creontiades-pallidus-1436425945-b7752.pdf
[5] ukmoths.org.uk
[6] Anikin, V. V., Sachkov, S. A., Zolotuhin, V. V. & Ustiuzhanin, P. Y., (2003). Fauna Lepidopterologica Volgo-Uralensis·' 150 years later: changes and additions. Part 7. Pyrales et Pterophores (Insecta, Lepidoptera). Atalanta 34(1/2), 223–250
[7] Waring, P & Townsend, M. Field Guide to the Moths of Great Britain and Ireland. 2003. British Wildlife Publishing, Hook Hampshire
[8] www.pyrgus.de
[9] García-Marí, F. (2012). PLAGAS DE LOS CÍTRICOS, Gestión Integrada en países de clima mediterráneo. Valencia, España, ed: Phytoma
[10] inaturalist.ca
[11] Kuchlein, J. H. (1978). Synopsis of the new european microlepidoptera with special reference to the ecology and taxonomy of the dutch species. Part 1. Introduction and Pyralidae (Galleriinae).
[12] Sociedad Gaditana de Historia Natural. (2021). VI BIOBLITZ de la SGHN Marisma de Trebujena (Cádiz). 22 y 23 de mayo de 2021. Sociedad Gaditana de Historia Natural, 9, 20-32
[13] Cid, N., Ibáñez, C., & Prat, N. (2008). Life history and production of the burrowing mayfly *Ephoron virgo*(Olivier, 1791) (Ephemeroptera:Polymitarcyidae) in the lower Ebro river: a comparison after 18 years. *Aquatic insects*, 30(3), 163-178.
