# Supplementary material for: Aerospace-foraging bats eat seasonably across varying habitats
Source: Sci Rep. 2023 Nov 10;13:19576. doi: 10.1038/s41598-023-46939-7 (PMC10638376; doi:10.1038/s41598-023-46939-7)
Supplement: Supplementary file 4 — Supplementary Information 4. [file 41598_2023_46939_MOESM4_ESM.pdf]

Title: Aerospace-foraging bats eat seasonably across varying habitats: implications for ecosystem services.

Authors: Joxerra AIHARTZA[1], Nerea VALLEJO[1], Miren ALDASORO[1], Juan L GARCIA-MUDARRA[2], Urtzi GOITI[1], Jesus NOGUERAS[2], Carlos IBAÑEZ[2]

Affiliations:

[1] Dpt. of Zoology and Animal cell Biology, University of the Basque Country UPV/EHU. Sarriena s/n, E48940, Leioa, The Basque Country.

[2] Estación Biológica de Doñana (CSIC), P.O. Box 1056, E41080, Sevilla, Spain.

Corresponding author: Joxerra Aihartza, joxerra.aihartza@ehu.eus;

Supplementary Material 5:

Insect species preyed upon by *Miniopterus schreibersii* considered as major and minor pests by the Spanish Ministry of Agriculture, arranged in order to the maximum seasonal consumption in the diet, measured as weighted percentage of occurrences: Max wPOO, maximum weighted percentage of occurrences (%) seasonally recorded; Max FOO, the maximum frequency of occurrences (%) seasonally recorded; Date, the sampling season the Max WPOO was recorded.

Source: Gil ÁM, de Ojer JLRS, Pérez MR, Ministerio de Agricultura (2014). Guía de gestión integrada de plagas. *https://www.mapa.gob.es/es/agricultura/temas/sanidad-vegetal/productos-fitosanitarios/guias-gestion-plagas/*

Species	Max wPOO	Max FOO	Date	Major Pest of:	Minor Pest of:
Agrotis segetum	17.8	90	24.09.18	Artichoque, cardoon; Horticultural: spinach, lettuce, swiss chard, escarole, starflower; zea mays	Cotton; Solanaceae: aubergine, pepper, tomato; Brassicas: broccoli, cauliflower, Brusels sprouts, cabbage, collard greens, Roman cauliflower; alfalfa; vineyards
Agrotis ipsilon	16.2	100	05.11.18	Artichoque, cardoon; Horticultural: spinach, lettuce, swiss chard, escarole, starflower; zea mays	Solanaceae: aubergine, pepper, tomato; Brassicas: broccoli, cauliflower, Brusels sprouts, cabbage, collard greens, roman cauliflower; alfalfa; vineyards
Creontiades pallidus	14.8	70	24.09.18	Solanaceae: aubergine, pepper, tomato; cotton	
Noctua pronuba	14.8	100	11.05.18		Vineyards
Autographa gamma	13.8	90	11.05.18	Horticultural: spinach, lettuce, swiss chard, escarole, starflower;	Brassicas: broccoli, cauliflower, Brusels sprouts, cabbage, collard greens, roman

					cauliflower; artichoque, cardoon; alfalfa; vineyards;
Prays citri	12.1	60	21.05.18	Citrus plants	
Thaumetopoea pityocampa	10.3	90	27.08.18	Conifers	
Peridroma saucia	7.7	60	11.05.18		Alfalfa
Palpita vitrealis	6.9	40	13.08.18	Olive groves	
Spodoptera exigua	5.0	30	24.09.18	Horticultural: spinach, lettuce, swiss chard, escarole, starflower; Solanaceae: aubergine, pepper, tomato;	Alfalfa; artichoque, cardoon; cotton;
Spodoptera littoralis	5.0	60	08.10.18	Horticultural: Spinach, lettuce, swiss chard, escarole, starflower; Solanaceae: aubergine, pepper, tomato; Rice;	Alfalfa; artichoque, cardoon; cotton;
Cydia fagiglandana	4.7	50	05.11.18		Castanea sativa; *Quercus sp.*;
Agrotis puta	4.4	50	08.10.18	Artichoque, cardoon; Horticultural: spinach, lettuce, swiss chard, escarole, starflower; zea mays	Solanaceae: aubergine, pepper, tomato; Brassicas: broccoli, cauliflower, Brusels sprouts, cabbage, collard greens, roman cauliflower; alfalfa; vineyards
Chrysodeixis chalcites	3.3	20	16.07.18		Brassicas: broccoli, cauliflower, Brusels sprouts, cabbage, collard greens, roman cauliflower;
Helicoverpa armigera	3.3	40	16.07.18	Artichoque, cardoon; Solanaceae: aubergine, pepper, tomato; cotton; zea mays; horticultural: spinach, lettuce, swiss chard, escarole, starflower; Brassicas: broccoli,	Alfalfa

				cauliflower, Brussels sprouts, cabbage, collard greens, roman cauliflower;	
Agrotis spinifera	2.5	11	10.09.18	Artichoque, cardoon; Horticultural: Spinach, lettuce, swiss chard, escarole, starflower; Zea Mays	Solanaceae: aubergine, pepper, tomato; Brassicas: Brocoli, Cauliflower, Brusels sprouts, Cabage, Collard greens, Romanescu; Alfalfa; Vineyards
Sesamia nonagrioides	2.5	10	24.09.18	Zea mays	
Prays oleae	2.5	30	13.08.18	Olive groves	
Noctua comes	2.2	20	27.08.18		Vineyards
Tuta absoluta	2.0	20	27.08.18	Solanaceae: aubergine, pepper, tomato;	
Pectinophora gossypiella	1.7	10	18.06.18	Cotton	
Plutella xylostella	1.4	10	02.07.18	Brassicas: Brocoli, Cauliflower, Brusels sprouts, Cabage, Collard greens, Romanescu;	
Agrotis trux	1.3	20	08.10.18	Artichoque, cardoon; Horticultural: Spinach, lettuce, swiss chard, escarole, starflower; Zea Mays	Solanaceae: aubergine, pepper, tomato; Brassicas: Brocoli, Cauliflower, Brusels sprouts, Cabage, Collard greens, Romanescu; Alfalfa; Vineyards
Earias insulana	1.3	20	08.10.18	Cotton	
Dioryctria mendacella	1.3	10	04.06.18		Conifers
Euzophera pinguis	1.3	10	21.05.18	Olive groves	
Agrotis bigramma	0.6	10	30.07.18	Artichoque, cardoon; Horticultural: Spinach, lettuce, swiss chard, escarole, starflower; Zea Mays	Solanaceae: aubergine, pepper, tomato; Brassicas: Brocoli, Cauliflower, Brusels sprouts, Cabage, Collard

					greens, Romanescu; Alfalfa; Vineyards
Cydia splendana	0.6	10	10.09.18	Quercus sp.;	Castanea sativa;
Pammene fasciana	0.6	11	08.10.18		Castanea sativa;
