# Supplementary material for: Aerospace-foraging bats eat seasonably across varying habitats
Source: Sci Rep. 2023 Nov 10;13:19576. doi: 10.1038/s41598-023-46939-7 (PMC10638376; doi:10.1038/s41598-023-46939-7)
Supplement: Supplementary file 5 — Supplementary Information 5. [file 41598_2023_46939_MOESM5_ESM.pdf]

Title: Aerospace-foraging bats eat seasonably across varying habitats: implications for ecosystem services.

Authors: Joxerra AIHARTZA[1], Nerea VALLEJO[1], Miren ALDASORO[1], Juan L GARCIA-MUDARRA[2], Urtzi GOITI[1], Jesus NOGUERAS[2], Carlos IBAÑEZ[2]

Affiliations:

[1] Dpt. of Zoology and Animal cell Biology, University of the Basque Country UPV/EHU. Sarriena s/n, E48940, Leioa, The Basque Country.

[2] Estación Biológica de Doñana (CSIC), P.O. Box 1056, E41080, Sevilla, Spain.

Corresponding author: Joxerra Aihartza, joxerra.aihartza@ehu.eus;

Supplementary Material 5:

List of species identified in the metabarcoding analysis but discarded for further analyses because they were previously recorded neither in the Iberian Peninsula nor within the 1000 km range in North Africa.

**Order**	**Family**	**Species**
Coleoptera	Cerambycidae	*Arhopalus tristis*
Coleoptera	Hydrophilidae	*Cercyon laminatus*
Coleoptera	Ptinidae	*Gastrallus knizeki*
Diptera	Chironomiidae	*Chironomus transvaalensis*
Diptera	Culicidae	*Culex peregrinus*
Diptera	Tachinidae	*Pandelleia albipennis*
Diptera	Tachinidae	*Phryno vetula*
Diptera	Tachinidae	*Phryxe pecosensis*
Hemiptera	Rhyparochromidae	*Remaudiereana nigriceps*
Hymenoptera	Ichneumonidae	*Netelia fuscicarpa*
Hymenoptera	Perilampidae	*Perilampus tristis*
Lepidoptera	Coleophoridae	*Coleophora spinella*
Lepidoptera	Coleophoridae	*Coleophora staehelinella*
Lepidoptera	Coleophoridae	*Coleophora telonica*
Lepidoptera	Tineidae	*Morophaga choragella*
Lepidoptera	Tortricidae	*Cochylimorpha meridiana*
Lepidoptera	Tortricidae	*Cochylis sannitica*
