# Supplementary material for: Aerospace-foraging bats eat seasonably across varying habitats
Source: Sci Rep. 2023 Nov 10;13:19576. doi: 10.1038/s41598-023-46939-7 (PMC10638376; doi:10.1038/s41598-023-46939-7)
Supplement: Supplementary file 6 — Supplementary Information 6. [file 41598_2023_46939_MOESM6_ESM.pdf]

Title: Aerospace-foraging bats eat seasonably across varying habitats: implications for ecosystem services.

Authors: Joxerra AIHARTZA[1], Nerea VALLEJO[1], Miren ALDASORO[1], Juan L GARCIA-MUDARRA[2], Urtzi GOITI[1], Jesus NOGUERAS[2], Carlos IBAÑEZ[2]

Affiliations:

[1] Dpt. of Zoology and Animal cell Biology, University of the Basque Country UPV/EHU. Sarriena s/n, E48940, Leioa, The Basque Country.

[2] Estación Biológica de Doñana (CSIC), P.O. Box 1056, E41080, Sevilla, Spain.

Corresponding author: Joxerra Aihartza, joxerra.aihartza@ehu.eus;

Supplementary Material 6:

Full list of arthropod species consumed by Miniopterus schreibersii, in accordance to the DNA extracted and amplified from their feces, including their frequencies of occurrences, FOO (%), percentage of occurrences, POO (%), and weighted percentages of occurrences, wPOO (%).

Order	Family	Species	FOO	POO	wPOO
Araneae	Tetragnathidae	*Metellina merianae*	0.71	0.09	0.02
Blattodea	Ectobiidae	*Loboptera decipiens*	0.71	0.09	0.04
Coleoptera	Carabidae	*Bembidion varium*	0.71	0.09	0.03
Coleoptera	Cerambycidae	*Arhopalus ferus*	0.71	0.09	0.06
Coleoptera	Mycetophagidae	*Typhaea stercorea*	1.43	0.18	0.17
Diptera	Anthomyiidae	*Delia platura*	2.14	0.27	0.14
Diptera	Chironomidae	*Tanytarsus eminulus*	0.71	0.09	0.03
Diptera	Culicidae	*Culex pipiens*	15.71	2.01	1.25
Diptera	Culicidae	*Culex theileri*	2.86	0.37	0.29
Diptera	Culicidae	*Culiseta longiareolata*	1.43	0.18	0.08
Diptera	Culicidae	*Ochlerotatus caspius*	2.14	0.27	0.15
Diptera	Drosophilidae	*Drosophila suzukii*	12.14	1.56	0.87
Diptera	Drosophilidae	*Leucophenga maculata*	12.86	1.65	1.15
Diptera	Limoniidae	*Dicranomyia ventralis*	7.86	1.01	1.61
Diptera	Limoniidae	*Gonomyia tenella*	2.14	0.27	0.09
Diptera	Limoniidae	*Limonia nubeculosa*	24.29	3.11	1.81
Diptera	Limoniidae	*Symplecta hybrida*	0.71	0.09	0.08
Diptera	Limoniidae	*Symplecta pilipes*	20.00	2.56	3.63
Diptera	Limoniidae	*Symplecta stictica*	2.86	0.37	0.21
Diptera	Phoridae	*Megaselia rufipes*	6.43	0.82	0.35
Diptera	Psychodidae	*Mormia tenebrosa*	0.71	0.09	0.05
Diptera	Psychodidae	*Paramormia ustulata*	2.86	0.37	0.37
Diptera	Psychodidae	*Philosepedon humeralis*	0.71	0.09	0.02
Diptera	Psychodidae	*Psychoda albipennis*	13.57	1.74	1.11
Diptera	Psychodidae	*Psychoda alternata*	1.43	0.18	0.31
Diptera	Syrphidae	*Episyrphus balteatus*	0.71	0.09	0.04
Diptera	Tachinidae	*Chetogena acuminata*	0.71	0.09	0.04
Diptera	Tachinidae	*Ligeria angusticornis*	0.71	0.09	0.02

Diptera	Tachinidae	*Pales pavida*	5.71	0.73	0.31
Diptera	Tachinidae	*Phryxe erythrostoma*	0.71	0.09	0.06
Diptera	Tachinidae	*Voria ruralis*	0.71	0.09	0.18
Diptera	Tephritidae	*Acanthiophilus helianthi*	0.71	0.09	0.24
Diptera	Tipulidae	*Tipula lateralis*	0.71	0.09	0.04
Ephemeroptera	Caenidae	*Caenis luctuosa*	30.00	3.84	3.54
Ephemeroptera	Leptophlebiidae	*Choroterpes picteti*	26.43	3.39	2.20
Ephemeroptera	Polymitarcidae	*Ephoron virgo*	29.29	3.75	2.48
Hemiptera	Aphrophoridae	*Neophilaenus campestris*	0.71	0.09	0.12
Hemiptera	Lygaeidae	*Nysius cymoides*	1.43	0.18	0.09
Hemiptera	Miridae	*Creontiades pallidus*	22.86	2.93	3.61
Hemiptera	Miridae	*Taylorigygus apicalis*	0.71	0.09	0.05
Lepidoptera	Bedelliidae	*Bedellia somnulentella*	0.71	0.09	0.04
Lepidoptera	Blastobasidae	*Blastobasis phycidella*	2.14	0.27	0.16
Lepidoptera	Choreutidae	*Tebenna micalis*	0.71	0.09	0.04
Lepidoptera	Cosmopterigidae	*Pyroderces argyrogrammos*	4.29	0.55	0.43
Lepidoptera	Cossidae	*Phragmataecia castaneae*	0.71	0.09	0.10
Lepidoptera	Crambidae	*Antigastra catalaunensis*	0.71	0.09	0.04
Lepidoptera	Crambidae	*Catoptria fulgidella*	2.86	0.37	0.20
Lepidoptera	Crambidae	*Chrysocrambus linetella*	0.71	0.09	0.10
Lepidoptera	Crambidae	*Eudonia lineola*	0.71	0.09	0.10
Lepidoptera	Crambidae	*Herpetogramma licarsisalis*	0.71	0.09	0.02
Lepidoptera	Crambidae	*Mecyna asinais*	0.71	0.09	0.05
Lepidoptera	Crambidae	*Nomophila noctuella*	6.43	0.82	1.22
Lepidoptera	Crambidae	*Palpita vitrealis*	10.00	1.28	1.50
Lepidoptera	Crambidae	*Spoladea recurvalis*	1.43	0.18	0.09
Lepidoptera	Crambidae	*Thopeutis galleriellus*	20.00	2.56	7.03
Lepidoptera	Crambidae	*Udea ferrugalis*	3.57	0.46	0.55
Lepidoptera	Crambidae	*Uresiphita gilvata*	3.57	0.46	0.58
Lepidoptera	Erebidae	*Autophila dilucida*	0.71	0.09	0.12
Lepidoptera	Erebidae	*Coscinia cribraria*	1.43	0.18	0.36
Lepidoptera	Erebidae	*Cymbalophora pudica*	0.71	0.09	0.06
Lepidoptera	Erebidae	*Dysgonia algira*	0.71	0.09	0.14
Lepidoptera	Erebidae	*Eublemma parva*	0.71	0.09	0.03
Lepidoptera	Erebidae	*Nodaria nodosalis*	2.14	0.27	0.22
Lepidoptera	Erebidae	*Odice jucunda*	1.43	0.18	0.09
Lepidoptera	Erebidae	*Odice pergrata*	2.14	0.27	0.32
Lepidoptera	Erebidae	*Pechipogo plumigeralis*	0.71	0.09	0.07
Lepidoptera	Gelechiidae	*Anacampsis populella*	0.71	0.09	0.04
Lepidoptera	Gelechiidae	*Aproaerema anthyllidella*	1.43	0.18	0.18
Lepidoptera	Gelechiidae	*Eulamprotes nigromaculella*	0.71	0.09	0.18
Lepidoptera	Gelechiidae	*Gelechia turpella*	0.71	0.09	0.02
Lepidoptera	Gelechiidae	*Pectinophora gossypiella*	0.71	0.09	0.12
Lepidoptera	Gelechiidae	*Phthorimaea operculella*	0.71	0.09	0.14

Lepidoptera	Gelechiidae	*Platyedra subcinerea*	2.86	0.37	0.46
Lepidoptera	Gelechiidae	*Scrobipalpa salinella*	0.71	0.09	0.24
Lepidoptera	Gelechiidae	*Tuta absoluta*	1.43	0.18	0.14
Lepidoptera	Geometridae	*Chiasmia aestimaria*	0.71	0.09	0.04
Lepidoptera	Geometridae	*Costaconvexa polygrammata*	1.43	0.18	0.24
Lepidoptera	Geometridae	*Ekboarmia atlanticaria*	0.71	0.09	0.09
Lepidoptera	Geometridae	*Eupithecia centaureata*	0.71	0.09	0.05
Lepidoptera	Geometridae	*Gymnoscelis rufifasciata*	17.14	2.20	3.35
Lepidoptera	Geometridae	*Idaea mustelata*	0.71	0.09	0.05
Lepidoptera	Geometridae	*Isturgia pulinda*	1.43	0.18	0.11
Lepidoptera	Geometridae	*Orthonama obstipata*	4.29	0.55	1.10
Lepidoptera	Geometridae	*Peribatodes ilicaria*	3.57	0.46	0.22
Lepidoptera	Geometridae	*Phaiogramma faustinata*	0.71	0.09	0.04
Lepidoptera	Geometridae	*Rhodometra sacraria*	7.14	0.91	0.77
Lepidoptera	Geometridae	*Selidoseam taeniolaria*	4.29	0.55	0.20
Lepidoptera	Geometridae	*Xanthorhoe ferrugata*	1.43	0.18	0.11
Lepidoptera	Geometridae	*Zernyia granataria*	1.43	0.18	0.06
Lepidoptera	Hepialidae	*Triodia sylvina*	6.43	0.82	0.48
Lepidoptera	Lasiocampidae	*Dendrolimus pini*	0.71	0.09	0.06
Lepidoptera	Lasiocampidae	*Lasiocampa trifolii*	2.14	0.27	0.16
Lepidoptera	Lasiocampidae	*Streblote panda*	1.43	0.18	0.15
Lepidoptera	Lecithoceridae	*Eurodachtha canigella*	0.71	0.09	0.02
Lepidoptera	Lecithoceridae	*Homaloxestis briantiella*	0.71	0.09	0.04
Lepidoptera	Noctuidae	*Aedia leucomelas*	0.71	0.09	0.09
Lepidoptera	Noctuidae	*Agrotis bigramma*	0.71	0.09	0.04
Lepidoptera	Noctuidae	*Agrotis ipsilon*	25.00	3.20	4.84
Lepidoptera	Noctuidae	*Agrotis puta*	3.57	0.46	0.31
Lepidoptera	Noctuidae	*Agrotis segetum*	37.14	4.76	5.39
Lepidoptera	Noctuidae	*Agrotis spinifera*	3.57	0.46	0.45
Lepidoptera	Noctuidae	*Agrotis trux*	3.57	0.46	0.22
Lepidoptera	Noctuidae	*Amphipyra cinnamomea*	0.71	0.09	0.03
Lepidoptera	Noctuidae	*Anarta sodae*	1.43	0.18	0.42
Lepidoptera	Noctuidae	*Anarta trifolii*	0.71	0.09	0.14
Lepidoptera	Noctuidae	*Apamea epomidion*	2.14	0.27	0.12
Lepidoptera	Noctuidae	*Autographa gamma*	25.71	3.29	3.48
Lepidoptera	Noctuidae	*Calophasia platyptera*	0.71	0.09	0.03
Lepidoptera	Noctuidae	*Chrysodeixis chalcites*	2.86	0.37	0.55
Lepidoptera	Noctuidae	*Conistra ligula*	0.71	0.09	0.05
Lepidoptera	Noctuidae	*Cryphia pallida*	0.71	0.09	0.03
Lepidoptera	Noctuidae	*Ctenoplusia limbirena*	0.71	0.09	0.14
Lepidoptera	Noctuidae	*Cucullia calendulae*	1.43	0.18	0.23
Lepidoptera	Noctuidae	*Dichagyris forcipula*	0.71	0.09	0.05
Lepidoptera	Noctuidae	*Euxoa wagneri*	4.29	0.55	0.25
Lepidoptera	Noctuidae	*Helicoverpa armigera*	7.14	0.91	0.97

Lepidoptera	Noctuidae	*Heliothis peltigera*	2.86	0.37	0.71
Lepidoptera	Noctuidae	*Hoplodrina hesperica*	0.71	0.09	0.03
Lepidoptera	Noctuidae	*Leucania loreyi*	25.71	3.29	3.64
Lepidoptera	Noctuidae	*Mythimna languida*	1.43	0.18	0.19
Lepidoptera	Noctuidae	*Mythimna vitellina*	22.86	2.93	2.91
Lepidoptera	Noctuidae	*Noctua comes*	3.57	0.46	0.45
Lepidoptera	Noctuidae	*Noctua pronuba*	22.86	2.93	2.99
Lepidoptera	Noctuidae	*Peridroma saucia*	22.14	2.84	3.04
Lepidoptera	Noctuidae	*Phlogophora meticulosa*	0.71	0.09	0.05
Lepidoptera	Noctuidae	*Plusia festucae*	1.43	0.18	0.24
Lepidoptera	Noctuidae	*Sesamia nonagrioides*	0.71	0.09	0.18
Lepidoptera	Noctuidae	*Spodoptera cilium*	3.57	0.46	0.33
Lepidoptera	Noctuidae	*Spodoptera exigua*	4.29	0.55	0.63
Lepidoptera	Noctuidae	*Spodoptera littoralis*	7.14	0.91	0.56
Lepidoptera	Noctuidae	*Thalpophila vitalba*	1.43	0.18	0.15
Lepidoptera	Noctuidae	*Thysanoplusia orichalcea*	0.71	0.09	0.05
Lepidoptera	Noctuidae	*Trichoplusia ni*	3.57	0.46	0.61
Lepidoptera	Noctuidae	*Xestia xanthographa*	1.43	0.18	0.08
Lepidoptera	Nolidae	*Earias insulana*	1.43	0.18	0.09
Lepidoptera	Nolidae	*Nola squalida*	5.71	0.73	2.13
Lepidoptera	Notodontidae	*Thaumetopoea pityocampa*	15.71	2.01	1.54
Lepidoptera	Oecophoridae	*Batia lambdella*	0.71	0.09	0.04
Lepidoptera	Oecophoridae	*Harpella forficella*	0.71	0.09	0.03
Lepidoptera	Plutellidae	*Plutella xylostella*	1.43	0.18	0.19
Lepidoptera	Praydidae	*Prays citri*	9.29	1.19	1.68
Lepidoptera	Praydidae	*Prays oleae*	9.29	1.19	0.76
Lepidoptera	Pterophoridae	*Amblyptilia acanthadactyla*	0.71	0.09	0.12
Lepidoptera	Pterophoridae	*Merrifieldia baliodactylus*	1.43	0.18	0.06
Lepidoptera	Pyralidae	*Apomyelois ceratoniae*	1.43	0.18	0.39
Lepidoptera	Pyralidae	*Bostra obsoletalis*	0.71	0.09	0.14
Lepidoptera	Pyralidae	*Cadra abstersella*	10.00	1.28	1.31
Lepidoptera	Pyralidae	*Cadra figulilella*	12.14	1.56	1.39
Lepidoptera	Pyralidae	*Cryptoblabes gnidiella*	7.86	1.01	1.21
Lepidoptera	Pyralidae	*Dioryctria mendacella*	1.43	0.18	0.14
Lepidoptera	Pyralidae	*Ematheudes punctella*	0.71	0.09	0.09
Lepidoptera	Pyralidae	*Epischnia illotella*	0.71	0.09	0.04
Lepidoptera	Pyralidae	*Euzophera pinguis*	0.71	0.09	0.09
Lepidoptera	Pyralidae	*Galleria mellonella*	2.86	0.37	0.27
Lepidoptera	Pyralidae	*Lamoria anella*	7.86	1.01	0.93
Lepidoptera	Pyralidae	*Oxybia transversella*	0.71	0.09	0.03
Lepidoptera	Pyralidae	*Phycitodes saxicola*	1.43	0.18	0.40
Lepidoptera	Tortricidae	*Crocidosema plebejana*	2.86	0.37	0.19
Lepidoptera	Tortricidae	*Cydia fagiglandana*	15.71	2.01	1.02
Lepidoptera	Tortricidae	*Cydia splendana*	0.71	0.09	0.04

Lepidoptera	Tortricidae	*Epinotia thapsiana*	1.43	0.18	0.06
Lepidoptera	Tortricidae	*Eucosma conterminana*	3.57	0.46	0.20
Lepidoptera	Tortricidae	*Gypsonoma aceriana*	1.43	0.18	0.08
Lepidoptera	Tortricidae	*Pammene fasciana*	0.71	0.09	0.04
Lepidoptera	Tortricidae	*Pseudargyrotoza conwagana*	1.43	0.18	0.17
Lepidoptera	Yponomeutidae	*Zelleria oleastrella*	1.43	0.18	0.18
Neuroptera	Myrmeleontidae	*Distoleon tetragrammicus*	0.71	0.09	0.04
Orthoptera	Gryllidae	*Eumodicogryllus bordigalensis*	0.71	0.09	0.18
Trichoptera	Lepidostomidae	*Lepidostoma hirtum*	0.71	0.09	0.03
